# Methyl jasmonate elicits distinctive hydrolyzable tannin, flavonoid, and phyto-oxylipin responses in pomegranate (*Punica granatum* L.) leaves

**DOI:** 10.1007/s00425-021-03735-9

**Published:** 2021-09-29

**Authors:** Lijing Chang, Sheng Wu, Li Tian

**Affiliations:** 1 Shanghai Key Laboratory of Plant Functional Genomics and Resources, Shanghai Chenshan Botanical Garden, Shanghai, 201602 China; 2 Shanghai Chenshan Plant Science Research Center, Chinese Academy of Sciences, Shanghai, 201602 China; 3 Department of Plant Sciences, Mail Stop 3, University of California, Davis, CA 95616 USA

**Keywords:** Methyl jasmonate, Flavonoid, Anthocyanin, Fatty acid, Phyto-oxylipin, Lipoxygenase

## Abstract

**Main conclusion:**

Transcriptome and biochemical analyses suggested that, while suppression of multiple flavonoids and anthocyanins occurs at least partially at the transcriptional level, increased biosynthesis of non-jasmonate phyto-oxylipins is likely controlled non-transcriptionally.

**Abstract:**

Methyl jasmonate (MeJA) produced in plants can mediate their response to environmental stresses. Exogenous application of MeJA has also shown to activate signaling pathways and induce phytoalexin accumulation in many plant species. To understand how pomegranate plants respond biochemically to environmental stresses, metabolite analysis was conducted in pomegranate leaves subjected to MeJA application and revealed unique changes in hydrolyzable tannins, flavonoids, and phyto-oxylipins. Additionally, transcriptome and real-time qPCR analyses of mock- and MeJA-treated pomegranate leaves identified differentially expressed metabolic genes and transcription factors that are potentially involved in the control of hydrolyzable tannin, flavonoid, and phyto-oxylipin pathways. Molecular, biochemical, and bioinformatic characterization of the only lipoxygenase with sustained, MeJA-induced expression showed that it is capable of oxidizing polyunsaturated fatty acids, though not located in the subcellular compartment where non-jasmonate (non-JA) phyto-oxylipins were produced. These results collectively suggested that while the broad suppression of flavonoids and anthocyanins is at least partially controlled at the transcriptional level, the induced biosynthesis of non-JA phyto-oxylipins is likely not regulated transcriptionally. Overall, a better understanding of how pomegranate leaves respond to environmental stresses will not only promote plant health and productivity, but also have an impact on human health as fruits produced by pomegranate plants are a rich source of nutritional compounds.

**Supplementary Information:**

The online version contains supplementary material available at 10.1007/s00425-021-03735-9.

## Introduction

When wounded or attacked by herbivores and pathogens, plants produce and emit methyl jasmonate (MeJA), which is perceived by non-wounded plant tissues and neighboring plants to activate defense response (Cheong and Choi 2003). Additionally, exogenous application of MeJA to a plant has shown to elicit signaling pathways as well as the production of pathogenesis-related proteins and defense chemicals known as phytoalexins. Phenolic phytoalexins, *e.g.* flavonoids and anthocyanins, have exhibited increased accumulation in response to MeJA treatment in different plants, such as *Arabidopsis thaliana*, grape (*Vitis vinifera*), banana (*Musa acuminata*), apple (*Malus domestica*), and red raspberry (*Rubus idaeus*) (Pandey et al. 2016; Portu et al. 2015; De Geyter et al. 2012; Shafiq et al. 2011; Flores and Ruiz del Castillo 2014). The biosynthesis of flavonoids and anthocyanins begins with the formation of naringenin chalcone from coumaroyl CoA and three molecules of malonyl CoA catalyzed by chalcone synthase (CHS) and the subsequent isomerization of naringenin chalcone to naringenin by chalcone isomerase (CHI). Naringenin is then used to generate the core flavonoid and anthocyanin skeletons, which are further modified with glycosyl, methyl, hydroxyl, and prenyl functional groups to give rise to diverse structures and functions (Tian et al. 2008).

In addition to phenolic phytoalexins, production of phyto-oxylipins upon MeJA induction has also been reported in a few plant species (Deboever et al. 2020). Phyto-oxylipins are oxygenated fatty acids and derivatives that play a role in plant growth, development, stress response, and innate immunity (Wasternack and Feussner 2018). The initial step of phyto-oxylipin biosynthesis involves oxidation of polyunsaturated fatty acids (PUFAs) to fatty acid hydroperoxides (HPOs) by lipoxygenases (LOXs) (Andreou and Feussner 2009). Plant LOXs are grouped into two subfamilies according to their protein sequences; type I LOXs are highly homologous (> 75% similarity) and do not contain a signal peptide, whereas type II LOXs possess an overall low sequence similarity (< 35%) but all contain a chloroplast target peptide (Feussner and Wasternack 2002). Plant LOXs can also be classified based on enzymatic activities; 9-LOXs and 13-LOXs target the C-9 and C-13 position of the fatty acid substrate, respectively (Feussner and Wasternack 2002). HPOs generated by LOXs can be further transformed to various phyto-oxylipins, such as hydroxy fatty acids by reductases, keto fatty acids by LOXs, epoxy fatty acids by peroxygenases (PXGs), and dihydroxy fatty acids by LOXs or α-dioxygenase α-DOXs). Notably, 13-hydroperoxy-linolenic acid (an HPO) produced from linolenic acid by 13-LOX can initiate a series of reactions to form jasmonic acid (JA), MeJA, and the bioactive JA-isoleucine conjugate.

Pomegranate (*Punica granatum* L.) is a specialty horticultural crop valued for the abundant phenolic compounds in its fruit, such as flavonoids, anthocyanins, and hydrolyzable tannins (HTs) that are derived from an intermediate of the shikimate pathway (Ono et al. 2016). Many studies have thus far focused on the role of pomegranate phenolics in alleviating stresses and illness in humans (Wu and Tian 2017). In contrast, little is known about the function of phenolics and other phytochemicals in defending pomegranate against abiotic and biotic factors (*e.g.* wounding, pathogens, MeJA induction) in leaves and fruits. Two recent reports evaluated pre-harvest MeJA treatment on the postharvest quality of pomegranate fruits (Koushesh Saba and Zarei 2019; García-Pastor et al. 2020). Total anthocyanins, flavonoids, and phenolics of MeJA-treated fruits, but not leaves, were analyzed collectively using a spectrophotometer. One of the studies also analyzed individual anthocyanins using mass spectrometry (García-Pastor et al. 2020). However, it remains unclear how pomegranate plant tissues, either leaves or fruits, respond to environmental stresses prior to fruit set.

To begin dissect interactions between pomegranate plants and environmental factors, we investigated the metabolic response of pomegranate leaves to exogenous MeJA application using high-performance liquid chromatography (HPLC) and liquid chromatography electrospray ionization tandem mass spectrometry (LC–ESI–MS/MS). Unique changes in HTs, flavonoids, and anthocyanins as well as alterations in lipids, fatty acids, and phyto-oxylipins were observed in pomegranate leaves treated with MeJA. Comparative transcriptome analysis, validated by real-time qPCR analysis, revealed that structural and/or regulatory genes involved in HT, flavonoid, anthocyanin, and phyto-oxylipin metabolism were differentially expressed in mock- and MeJA-treated pomegranate leaves. The only LOX gene that exhibited a sustained upregulated expression in MeJA-treated leaves was subjected to further molecular, biochemical, and phylogenetic characterization.

## Materials and methods

### Chemicals

The β-glucogallin and pentagalloylglucose standards were purchased from Shanghai Yuanye Bio-Technology Co., Ltd (Shanghai, China). Chemicals used in the LOX assay were obtained from the following vendors: 3-(dimethylamino)benzoic acid (DMAB) (Adamas Reagent, Co., Ltd., Shanghai, China), linoleic acid (Sigma-Aldrich, St. Louis, MO, USA), 3-methyl-2-benzothiazolinone (MBTH) and hemoglobin (Sangon Biotech Co., Ltd., Shanghai, China).

### Plant materials

Pomegranate fruits and seeds (cv. Wonderful) were generously provided by the Panzhihua Academy of Agricultural and Forest Sciences, and identified by Dr. Binjie Ge at Shanghai Chenshan Botanical Garden. A voucher specimen (No. CSH0173966) was deposited at the herbarium of Shanghai Chenshan Botanical Garden, Shanghai, China. Pomegranate seedlings were grown in a temperature-controlled growth room for 6 weeks at 25 °C and 16 h light/8 h dark. The MeJA concentration for spray application to plant tissues reportedly ranges from 100 to 250 μM (Ku et al. 2014; Hickman et al. 2017). Different concentrations of MeJA were initially applied to pomegranate leaves, of which 200 μM MeJA led to a discernable metabolic response in the preliminary analysis and was used for the metabolite and gene expression analyses described in this study. Prior to the MeJA treatment, half of the pomegranate plants were moved to another growth room with similar conditions. While plants in one growth room were sprayed with 200 μM MeJA, those in the other growth room were sprayed with water (i.e. mock control). At 2-h, 3-h, 6-h, 12-h, 24-h, 30-h, 36-h, 48-h, and 72-h after the treatment, leaves from 3 to 5 mock- or MeJA-treated plants were pooled, which constitute one biological replicate. Three biological replicates were collected for the mock- and MeJA-treatment experiments; each biological replicate was divided into aliquots for metabolite profiling and gene expression analyses.

### Metabolite profiling analysis

Pomegranate leaves were lyophilized, weighed, and ground into fine powder using zirconia beads in a bead beater (Mixer Mill MM 400, Retsch GmbH, Haan, Germany) for 90 s at 30 Hz. For HPLC analysis, the leaf sample was extracted in 70% methanol for 60 min under sonication and centrifuged at 13,000 rpm for 10 min. The supernatant was transferred to an HPLC vial, of which 30 μL was injected onto a reverse phase HPLC (Agilent 1200, Agilent Technologies, Santa Clara, CA, USA) and analyzed as previously described (Wilson et al. 2019). Metabolites were detected by UV absorption at 254 nm, 280 nm, 320 nm, and 360 nm. Standard calibration curves of β-glucogallin and pentagalloylglucose were constructed; they were used for converting the areas of peaks that match the retention times and absorption spectra of β-glucogallin and pentagalloylglucose to the respective concentrations.

For LC–ESI–MS/MS analysis, the homogenized leaf sample (100 mg) was extracted in 1 mL of 70% methanol at 4 °C overnight. On the following day, the methanolic extract was centrifuged at 10,000 × g for 10 min and the supernatant was passed through a CNWBOND Carbon-GCB SPE cartridge (ANPEL, Shanghai, China) and a 0.22-μm syringe filter (ANPEL) prior to metabolite analysis.

The extract (2 μL) was analyzed using LC–ESI–MS/MS (Shim-pack UFLC, Shimadzu, Kyoto, Japan; QTRAP 6500, Applied Biosystems, Foster City, CA, USA) and a reverse phase C_18_ column (ACQUITY UPLC HSS T3, 1.8 βm, 2.1 mm × 100 mm, Waters, Milford, MA, USA). Metabolites were eluted using solvents (A) water containing 0.04% acetic acid, and (B) acetonitrile containing 0.04% acetic acid at a gradient of 0–11 min, 95–5% A; 11–12 min, 5% A; 12–12.1 min, 5–95% A; 12.1–15 min, 95% A. The flow rate was maintained at 0.4 mL min^−1^. Linear ion trap (LIT) and triple quadrupole (QQQ) MS scans were acquired in positive- and negative-ion modes. The turbo spray ion source was operated at 500 °C with an ionization voltage of 5500 V. The ion source gas I, gas II, and curtain gas were set at 55 psi, 60 psi, and 25 psi, respectively. The collision gas (nitrogen) was set at 5 psi. For the MRM analysis, declustering potential (DP) and collision energy (CE) were optimized for each precursor-product ion transition.

For metabolite identification, the LC–ESI–MS/MS data were compared with an MS2T library of commercial standards and previously identified compounds published in mass spectral databases (when commercial standards are not available) (Chen et al. 2013). Pomegranate metabolites were annotated based on the retention times, accumulate *m/z* values, and fragmentation patterns that match the MS2T library entries (Chen et al. 2013). Metabolite quantification was performed using the MRM method as described by Dresen et al*.* (Dresen et al. 2010). The biological replicates of each treatment (mock or MeJA) were averaged for comparative metabolite analysis. The Variable Importance in Projection (VIP) value was obtained from the Orthogonal Partial Least Squares Discriminant Analysis (OPLS-DA) model. Metabolites with |Log_2_FC|> 1 and VIP >  = 1 were considered significantly changed.

### Transcriptome analysis

Total RNA was extracted from pomegranate leaves using TRIzol reagent (Invitrogen, Carlsbad, CA, USA) and quantified using Nanodrop2000 (ThermoFisher Scientific, Waltham, MA, USA). Integrity of the RNA samples was verified through separation on an agarose gel (no visible degradation) and determination of the O.D._260_/_280_ ratio (between 1.8 and 2.2) using Nanodrop2000. Enrichment of mRNA from total RNA was carried out using the oligo (dT) magnetic beads (Invitrogen). mRNA-Seq libraries were constructed using the Truseq RNA library preparation kit (Illumina, San Diego, CA, USA). Transcriptome analysis was conducted on Illumina HiSeq4000 and 55–60 million of 150-bp paired end reads (PE150) were obtained for each sample library.

The raw sequence data were processed by removing the adaptor sequences as well as short (< 50 bp), low quality (Q < 30), and polyN (> 10%) reads using SeqPrep (https://github.com/jstjohn/seqprep) and Sickle (https://github.com/najoshi/sickle). Over 95% of the cleaned reads were uniquely mapped to the reference pomegranate genome for each sample library using HISAT2 (Kim et al. 2015) and the mapped reads were a∆ssembled using StringTie (Pertea et al. 2015). The assembled transcriptome sequences were annotated using NCBI_NR (ftp://ftp.ncbi.nlm.nih.gov/blast/db/). Transcript abundance was determined by the RNA-Seq by Expectation Maximization (RSEM) method and expressed as Transcripts Per Kilobase Million (TPM) (Li and Dewey 2011). Differential gene expression analysis was performed using DESeq2 (Love et al. 2014), with a threshold of |log_2_FC|> 1 and adjusted *P* value < 0.05.

### Real-time qPCR analysis

Total RNA was extracted from mock- and MeJA-treated pomegranate leaves using the RNAprep Pure Plant Kit (Tiangen Biotech Co., Ltd., Beijing, China). Reverse transcription (RT) was performed using total RNA and the PrimeScript™ RT Reagent Kit (Takara Bio Inc., Kusatsu, Japan). Quantitative PCR (qPCR) was carried out using the TB Green™ Premix Ex Taq™ (Tli RNaseH Plus) kit (Takara) and a StepOnePlus Real-Time PCR System (ThermoFisher Scientific). Melting curve analysis was conducted and showed a single amplification product for each primer pair. For the RT-qPCR analysis, three biological replicates and each with three technical replicates were examined for mock- and MeJA-treated samples. Gene expression was analyzed using the comparative C_t_ (∆∆C_t_) method (Livak and Schmittgen 2001) and significance levels were determined using a two-tailed Student’s *t* test. The primer sequences for the real-time qPCR analysis and the amplification efficiencies of the primer pairs are shown in Table S1.

### Expression and purification of recombinant proteins and enzyme assays

The open reading frame of *Pgr025417* (encoding a putative LOX) was synthesized for optimal codon usage in *E. coli* (Genewiz, Suzhou, China) and cloned in pET28a. The recombinant plasmid was transformed into *E. coli* BL21 (DE3) cells. A 5-mL Luria Bertani (LB) culture with 50 μg mL^−1^ kanamycin was started from a single colony and incubated overnight with shaking at 37 °C. The overnight culture was used to inoculate a 100-mL LB medium with 50 μg mL^−1^ kanamycin and allowed to grow to an O.D._600_ of 0.5. Isopropyl-β-D-thiogalactoside (IPTG) was then added to a final concentration of 0.1 mM for induction of protein expression. After incubation with shaking at 16 °C for 18 h, the cells were harvested by centrifugation. The cell pellets were resuspended in the lysis buffer (50 mM NaH_2_PO_4_, pH 7.4, 300 mM NaCl, 10 mM imidazole) and homogenized using a cell disruptor (Constant Systems Ltd, Northants, UK). His-tagged proteins were purified using Ni–NTA beads (ThermoFisher Scientific) with the wash buffer (50 mM NaH_2_PO_4_, pH 7.4, 300 mM NaCl, 25 mM imidazole) and the elution buffer (50 mM NaH_2_PO_4_, pH 7.4, 300 mM NaCl, 500 mM imidazole). The purified proteins were separated on a 10% SDS-PAGE gel for visualization of protein purity. The concentration of the purified proteins was determined using the Bradford assay (Bradford 1976).

For the LOX assay, linoleic acid was used as substrate in a two-step, colorimetric method with slight modifications (Anthon and Barrett 2008). The 500-μL reaction mixture, including 50 mM Na-phosphate, pH 6, 10 mM DMAB, 0.5 mM linoleic acid, and various amounts of purified recombinant proteins (1.5 mg mL^−1^), was incubated at 25 °C for 10 min. A second solution (500 μL) containing 0.2 mM MBTH and 0.1 mg mL^−1^ hemoglobin was added to the reaction mixture, which was incubated for an additional 5 min. The reaction was terminated by adding 500 μL 1% (w/v) sodium lauryl sulfate. Light absorption at 598 nm was determined.

### Subcellular localization and phylogenetic analyses

A search of the annotated pomegranate genome (Qin et al. 2017) identified 11 putative full-length LOXs (786 aa to 970 aa), including Pgr025413, Pgr020032, Pgr025418, Pgr025417, Pgr018982, Pgr018980, Pgr016852 (full-length sequence in GenBank XP_031395793), Pgr009839, Pgr008562, Pgr025678, and Pgr013780. Subcellular localization and cleavage sites of signal peptides for the pomegranate LOXs were predicted using TargetP 2.0 (http://www.cbs.dtu.dk/services/TargetP/) (Almagro Armenteros et al. 2019).

For phylogenetic analysis, protein sequences of LOXs were aligned using Multiple Sequence Comparison by Log-Expectation (MUSCLE) (Edgar 2004). Neighbor-joining trees were constructed in Molecular Evolutionary Genetics Analysis (MEGA) X with 3,000 bootstrap repeats (Kumar et al. 2018). The GenBank accession numbers of the LOXs are: *At*LOX1 (Q06327), *At*LOX2 (P38418), *At*LOX3 (Q9LNR3), *At*LOX4 (Q9FNX8), *At*LOX5 (Q9LUW0), *At*LOX6 (Q9CAG3), *Gm*LOX1 (NP_001236153), *Gm*LOX2 (NP_001237685), *Gm*LOX3 (P09186), *Gm*LOX4 (P38417), *Gm*LOX5 (Q43446), *Gm*LOX6 (Q43440), *Hv*LOX1 (P29114), *Hv*LOX2.1 (P93184), *Sl*LOXA (NP_001234856), *Sl*LOXB (NP_001234873), *Sl*LOXD (NP_001307221), *Os*LOX (Q27PX2), *Os*LOX1 (Q76I22), *Os*LOX2 (P29250), *Os*LOX7 (P38419), *Os*LOX_RCI-1 (Q9FSE5), *Zm*LOX1 (NP_001105003), *Zm*LOX3 (NP_001105515), *Zm*LOX4 (NP_001105974), *Zm*LOX5 (NP_001105975), *Zm*LOX6, (NP_001105976), and *Zm*LOX9 (NP_001105977).

### TF-binding site analysis

To predict the binding sites of TFs, 1000 bp upstream of the ATG start codon of the target genes was obtained from GenBank and searched against the *Eucalyptus grandis* TFs in PlantRegMap (version 5) (Tian et al. 2020). The threshold value for binding site identification was set at *P* ≤ 1e-4.

### Statistical analysis

Statistical analysis for the metabolite quantification, transcriptome, and real-time qPCR data is described under the respective sections.

## Results

### MeJA modulates cell signaling and metabolic pathways in pomegranate leaves

To understand the genome-wide transcriptional response of pomegranate to MeJA elicitation, transcriptomes of pomegranate leaves at 2-h, 6-h, 24-h, and 72-h after MeJA or mock treatment (each with three biological replicates) were analyzed (Fig. S1). Approximately 55 million raw sequence reads (2 × 150 bp paired-end) were obtained for each transcriptome with GC content around 52% and Q30 values ranging from 91.6 to 95% (Table S2). For all transcriptomes, more than 96% of the cleaned sequence reads were mapped to the reference pomegranate genome (Qin et al. 2017) (Table S3). A majority of the assembled transcripts were less than 1000 bp (34.3%), 1000 bp—2000 bp (32.9%), or 2000 bp—3000 bp (18.1%) (Table S4).

To identify pathways that are significantly enriched with differentially expressed genes (DEGs) at the above-mentioned time points, genes that show significantly different expression (|log_2_FC|> 1, adjusted *P* < 0.05) between MeJA- and mock-treated leaves were compared to the Kyoto Encyclopedia of Genes and Genomes (KEGG) database (Fig. S1). Application of MeJA modulated the expression of genes in plant hormone and mitogen-activated protein kinase (MAPK) signaling pathways as well as fatty acid metabolism at all time points, with the only exception of those in plant hormone pathways at 24-h (Fig. S1). While changes in aromatic amino acid metabolic (including the shikimate pathway) genes became evident at 6-h after MeJA treatment (Fig. S1b), a surge of modified expression of flavonoid metabolic genes was observed for the 24-h and 72-h post-MeJA treatment leaves (Figs. S1c and S1d).

### Shikimate and HT pathway genes and HT metabolites were induced in MeJA-treated pomegranate leaves

**Fig. 1:**
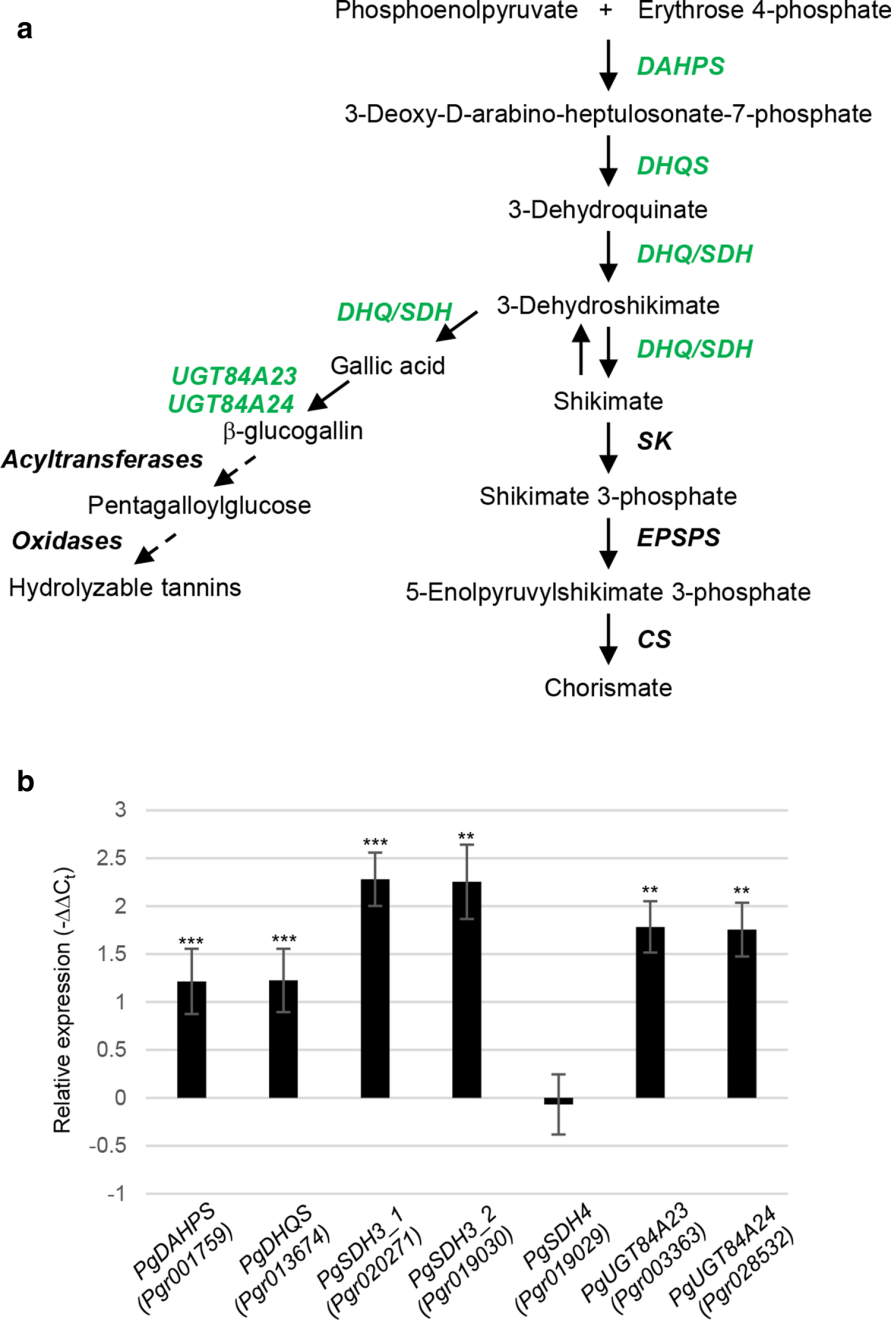
Expression of shikimate and hydrolyzable tannin (HT) pathway genes was induced by methyl jasmonate (MeJA) application. **a** A simplified diagram of shikimate and HT biosynthetic pathways. Genes exhibited increased expression upon MeJA elicitation in the 6-h transcriptomes are highlighted in green. Dashed arrows indicate multiple reaction steps. DAHPS, 3-deoxy-D-arabino-heptulosonate-7-phosphate synthase; DHQS, 3-dehydroquinate synthase; DHQ/SDH, 3-dehydroquinate dehydratase/shikimate dehydrogenase; SK, shikimate kinase; EPSPS, 5-enolpyruvylshikimate-3-phosphate synthase; CS, chorismate synthase. **b** Real-time qPCR analysis of differentially expressed shikimate and HT pathway genes identified in the transcriptome analysis. *P < 0.05; **P < 0.01; ***P < 0.001

As revealed in the transcriptome and KEGG pathway enrichment analysis, three shikimate biosynthetic pathway genes showed upregulated expression in MeJA-treated leaves relative to mock controls at 6-h, including *3-deoxy-D-arabino-heptulosonate-7-phosphate synthase (DAHPS)*, *3-dehydroquinate synthase (DHQS)*, and the bifunctional *3-dehydroquinate dehydratase/shikimate dehydrogenase* (*DHQ/SDH*; abbreviated as *SDH*) (Figs. S1b and 1a). In particular, three isoforms of pomegranate *SDHs* were identified and showed differential expression in the transcriptome analysis, *Pgr020271*, *Pgr019030*, and *Pgr019029*, which correspond to the previously designated *PgSDH3_1*, *PgSDH3_2*, and *PgSDH4*, respectively (Fig. 1a) (Habashi et al. 2019). An intermediate of the shikimate pathway, 3-dehydroshikimate, serves as a precursor for the synthesis of gallic acid, which is esterified to a glucose molecule to form β-glucogallin (Fig. 1a). In pomegranate, this committed step in HT biosynthesis is catalyzed by two glycosyltransferases *Pg*UGT84A23 and *Pg*UGT84A24 (Ono et al. 2016). As with the shikimate pathway genes, *PgUGT84A23* and *PgUGT84A24* also showed increased expression in the transcriptomes of MeJA-treated leaves at 6-h (Fig. 1a). The heightened expression of these shikimate and HT biosynthetic genes was validated by real-time qPCR analysis, with the exception of *PgSDH4* (*Pgr019029*) (Fig. 1b).

**Fig. 2:**
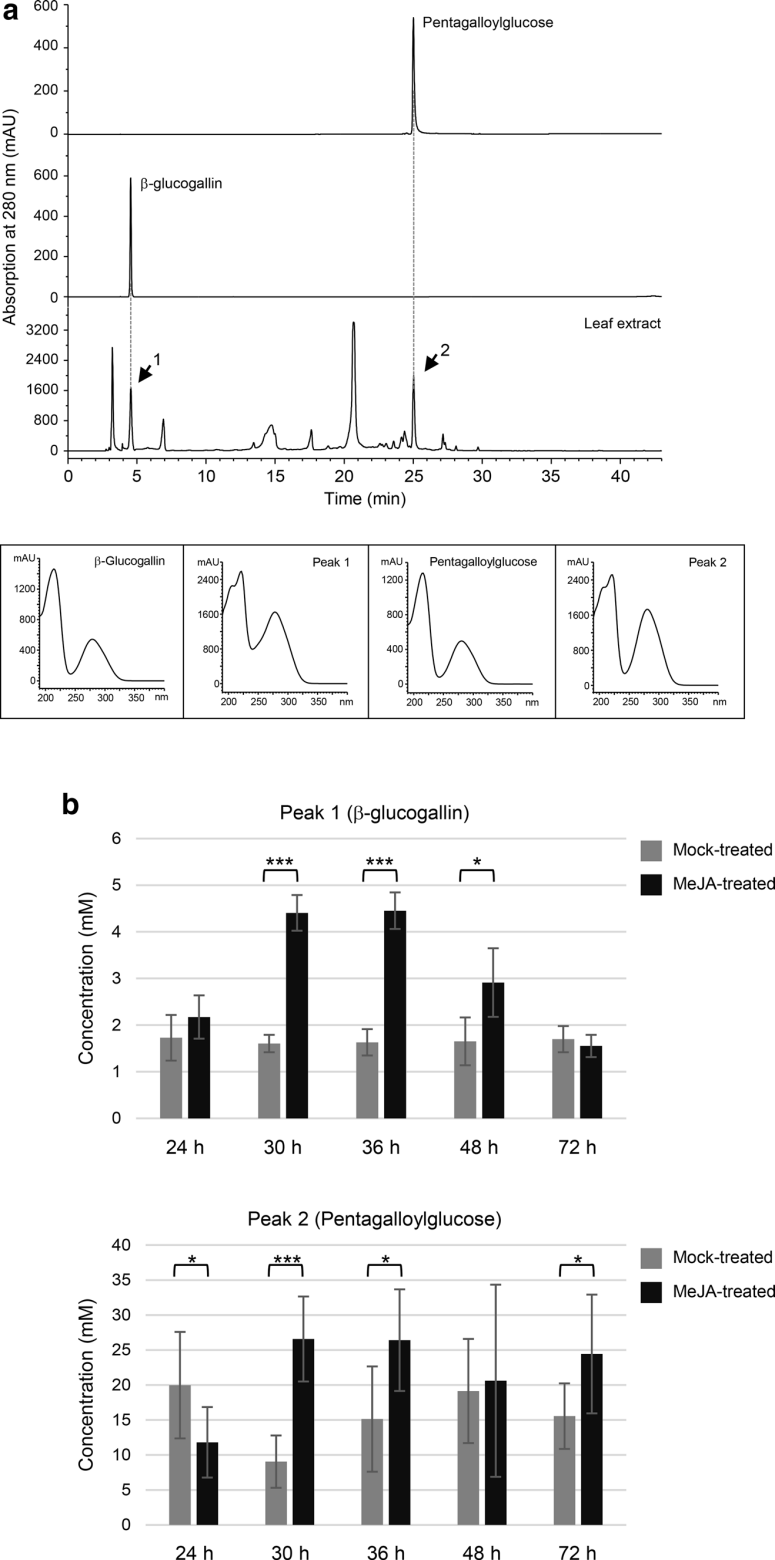
Hydrolyzable tannin accumulation was induced by methyl jasmonate (MeJA) application. **a** HPLC elution profiles of the authentic pentagalloylglucose and β-glucogallin standards and a representative pomegranate leaf extract of phenolic metabolites. The absorption spectra of pentagalloylglucose, β-glucogallin, and peaks 1 and 2 are shown. b Areas (mAU) of peaks 1 and 2 in the extracts of leaf samples harvested at 24-h, 30-h, 36-h, 48-h, and 72-h after MeJA- and mock-treatment. *P < 0.05; **P < 0.01; ***P < 0.001

To determine whether changes in the amount of shikimate and HT biosynthetic gene transcripts may affect the level of metabolites derived from these pathways, phenolic metabolites were extracted from leaves harvested at 24-h, 30-h, 36-h, 48-h, and 72-h after MeJA or mock application and analyzed by HPLC (Fig. 2). It should be noted that these time points were chosen to account for the time needed for protein synthesis and metabolite production and accumulation after the observed expression changes of shikimate and HT biosynthetic genes at 6-h. The retention times and absorption spectra of two metabolites eluted at 4.57 min (peak 1) and 24.98 min (peak 2) matched to those of the HT pathway intermediates β-glucogallin and pentagalloylglucose, respectively (Figs. 1a and 2a). Both peaks showed significant changes in integrated peak areas at multiple time points (Fig. 2b). Specifically, peak 1 increased in MeJA-treated leaves relative to mock controls at 30-h, 36-h, and 48-h (Fig. 2b). Interestingly, peak 2 in MeJA-treated leaves initially decreased at 24-h, but subsequently increased at 30-h and 36-h before returning to a level similar to mock controls at 48-h and 72-h (Fig. 2b).

### Reduction of most flavonoids and anthocyanins as well as increased methylated flavones and flavonols were apparent in MeJA-treated pomegranate leaves

To investigate whether exogenous application of MeJA may trigger broad scale metabolic changes in pomegranate, metabolite profiling analysis was conducted on leaves collected at 72-h after mock- or MeJA treatment using LC–ESI–MS/MS. Metabolite annotation and quantification were performed using an MS/MS spectral tag (MS2T) library and multiple reaction monitoring (MRM), respectively. Of the 658 metabolites that were detected, 29 showed increased and 73 exhibited decreased accumulation in MeJA-treated leaves compared to the mock controls (|Log_2_FC|> 1; Tables S5 and S6). For the differentially accumulated metabolites, there was an overall enrichment of metabolites involved in plant secondary/specialized metabolism (67 of 102), particularly phenolic compounds (63 of 102) (Table S6).

**Fig. 3:**
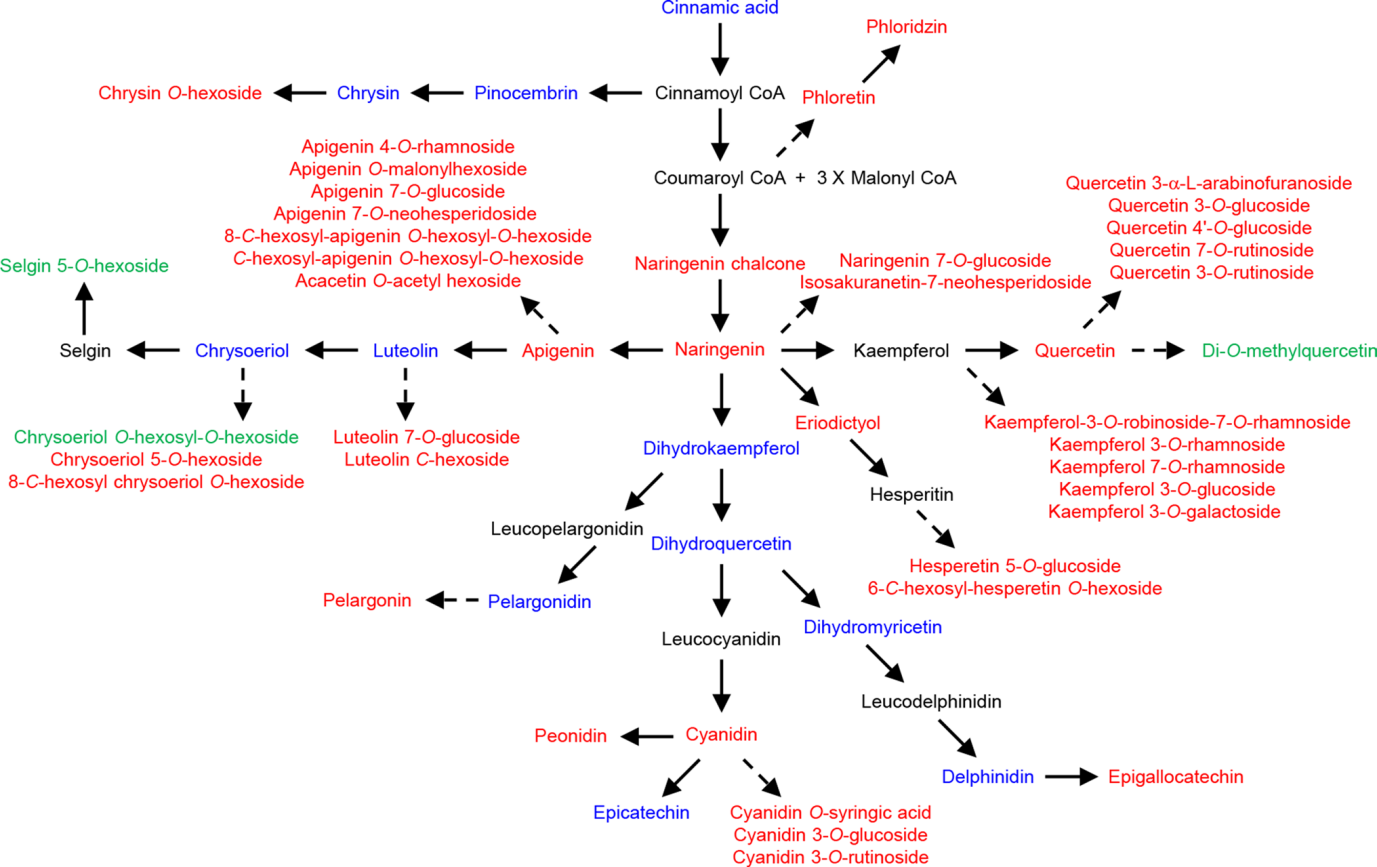
Flavonoid and anthocyanin levels were altered upon methyl jasmonate (MeJA) application. Metabolites with increased, decreased, and non-significantly changed accumulation (two-fold threshold) in MeJA-treated pomegranate leaves compared to mock-treated controls, harvested at 72-h post treatment, are colored in green, red, and blue, respectively. Pathway intermediates that were not detected in the liquid chromatography electrospray ionization tandem mass spectrometry analysis are shown in black. Dashed arrows indicate multiple reaction steps

Among phenolics, a concerted reduction in a wide range of flavonoids and anthocyanins (42 of the 73 decreased compounds) was apparent in MeJA-treated leaves (Fig. 3; Table S6). Intriguingly, three mono- or di-*O*-methylated flavones and flavonols, including di-*O*-methylquercetin, chrysoeriol *O*-hexosyl-*O*-hexoside, and selgin 5-*O*-hexoside, were increased in MeJA-treated leaves (Fig. 3; Table S6). Several intermediates of the flavonoid and anthocyanin pathways, including luteolin, chrysoeriol, dihydrokaempferol, dihydroquercetin, dihydromyricetin, epicatechin, delphinidin, and pelargonidin, were detectable, but did not show significant changes in MeJA-treated leaves (Fig. 3; Table S5). Hydroxycinnamoyl derivatives, isoflavones, and coumarins were among other phenolics that showed reduced accumulation upon MeJA induction (Table S6). By contrast, two phenolic acids, 2,3-dihydroxybenzoic acid and protocatechuic acid (3,4-dihydroxybenzoic acid), and a coumarin, 6-methylcoumarin, were increased in MeJA-treated leaves (Table S6).

**Fig. 4:**
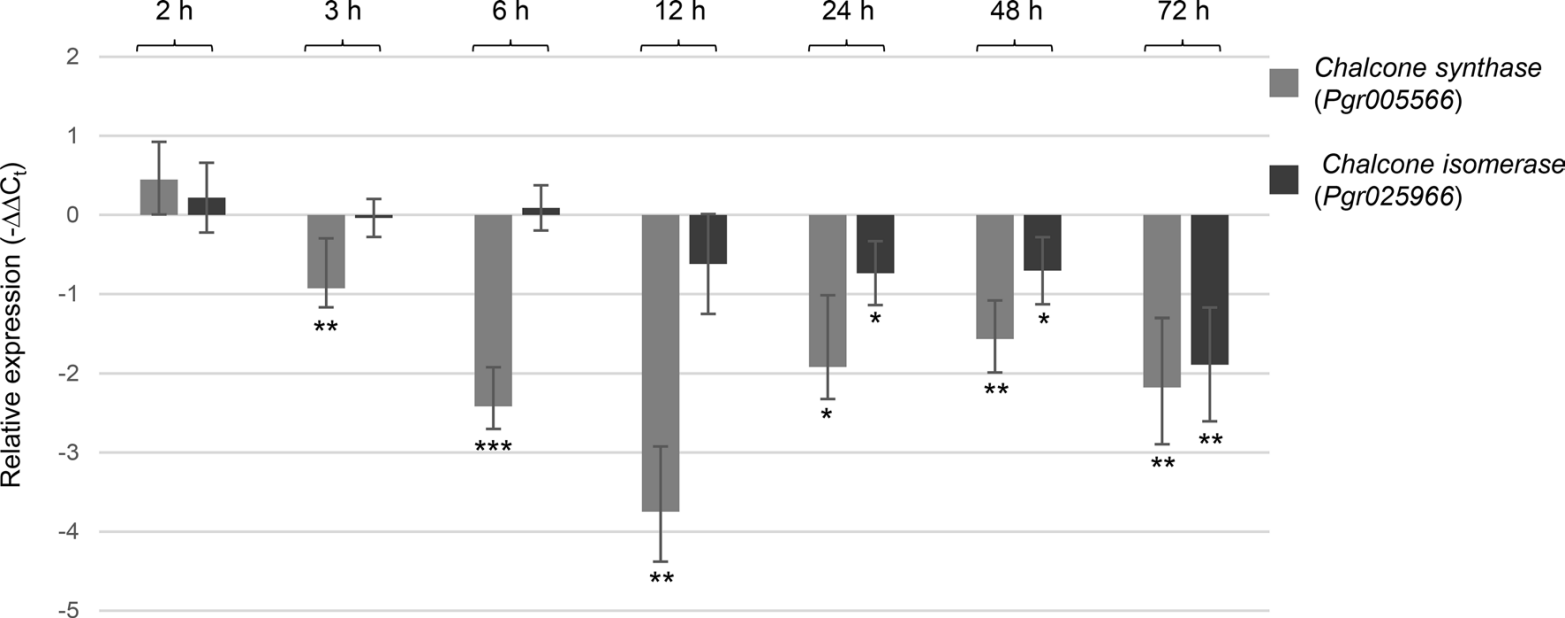
Relative expression (∆∆C_t_) of chalcone synthase and chalcone isomerase genes in leaf samples harvested at 2-h, 3-h, 6-h, 12-h, 24-h, 48-h, and 72-h after MeJA- and mock-treatment

Consistent with the largely reduced flavonoids and anthocyanins in MeJA-treated pomegranate leaves, the transcripts of two key enzymes for flavonoid and anthocyanin biosynthesis, CHS (Pgr005566) and CHI (Pgr025966), were significantly decreased at 6-h and 24-h after MeJA application according to the transcriptome analysis (Fig. 4). Real-time qPCR analysis was carried out to examine *CHS* and *CHI* expression with additional time points, including 2-h, 3-h, 6-h, 12-h, 24-h, 48-h, and 72-h (Fig. 4). *CHS* transcripts dropped in MeJA-treated leaves at 3-h and remained to be significantly lower than those in mock controls until 72-h, with the largest decrease at 12-h. In contrast, reduction in *CHI* expression was only significant at 24-h, 48-h, and 72-h post-MeJA treatment (Fig. 4).

### Lipids were converted to free fatty acids and phyto-oxylipins in MeJA-treated pomegranate leaves

**Fig. 5:**
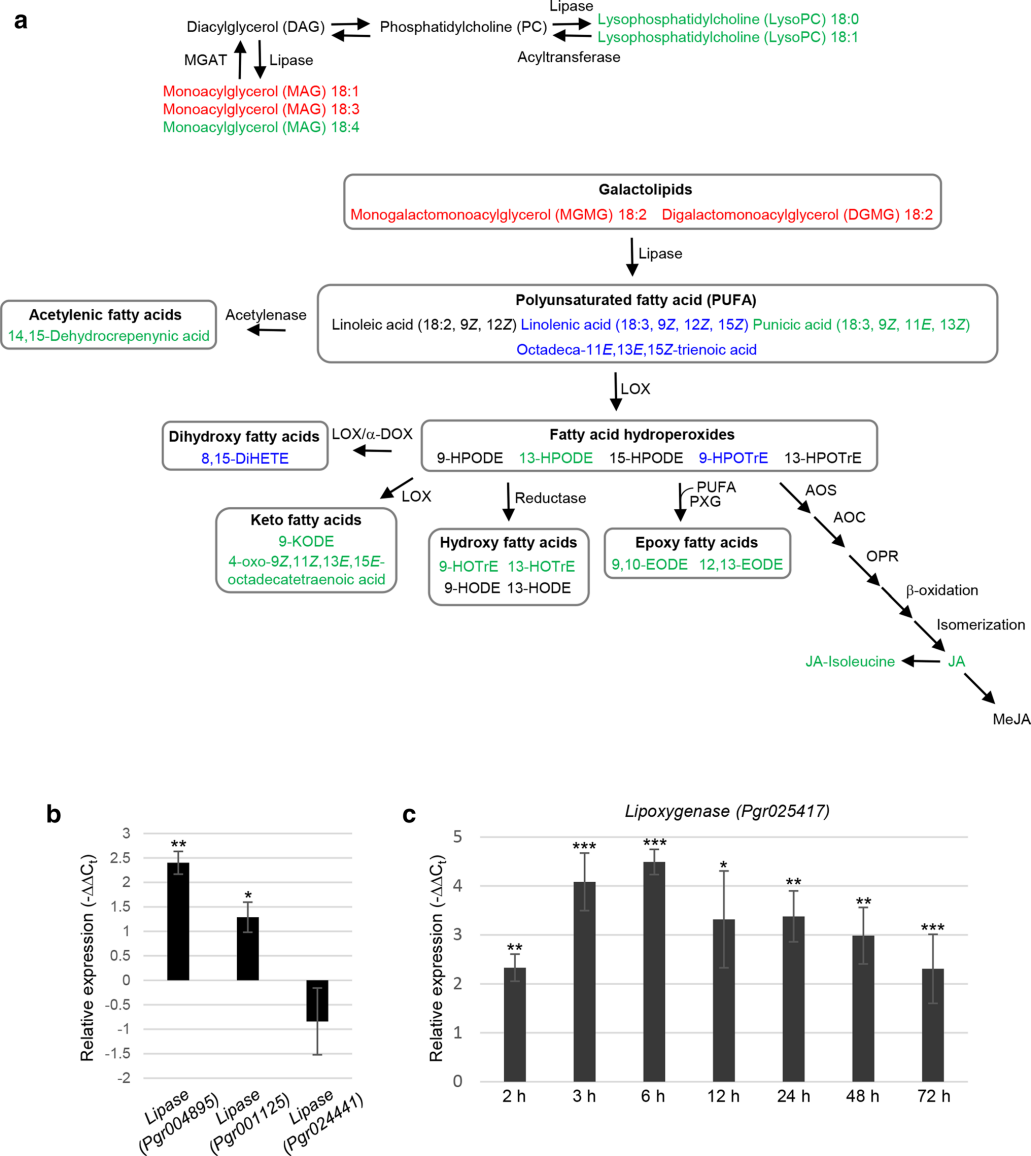
Methyl jasmonate (MeJA) application led to modifications in phyto-oxylipin metabolism. **a** Metabolites with increased, decreased, and non-significantly changed accumulation (two-fold threshold) in MeJA-treated pomegranate leaves compared to mock-treated controls, harvested at 72-h post treatment, are colored in green, red, and blue, respectively. Pathway intermediates that were not detected in the liquid chromatography electrospray ionization tandem mass spectrometry analysis are shown in black. *DiHETE* dihydroxyeicosatetraenoic acid, *EODE* epoxy octadecadienoic acid, *HODE* hydroxyoctadecadienoic acid, *HOTrE* hydroxyoctadecatrienoic acid, *HPODE* hydroperoxyoctadecadienoic acid, *HPOTrE* hydroperoxyoctadecatrienoic acid, *JA* jasmonic acid, *KODE* keto octadecadienoic acid, *LOX* lipoxygenase, *MeJA* methyl jasmonate, *AAPT* aminoalcohol aminophosphotransferase, *β-DOX* β-dioxygenase, *AOC* allene oxide cyclase, *AOS* allene oxide synthase, *LOX* lipoxygenase, *MGAT* monoacylglycerol acyltransferase, *OPR* oxo-phytodienoic acid (OPDA) reductase, *PXG* peroxygenase. **b** Relative expression (∆∆C_t_) of three putative lipase genes in leaf samples harvested at 24-h after MeJA- and mock-treatment. **c** Relative expression (∆∆C_t_) of a putative lipoxygenase gene (*Pgr025417*) in leaf samples harvested at 2-h, 3-h, 6-h, 12-h, 24-h, 48-h, and 72-h after MeJA- and mock-treatment. *P < 0.05; **P < 0.01; ***P < 0.001

Besides flavonoids and anthocyanins, changes in lipid molecules were also evident in pomegranate leaves at 72-h after MeJA induction (Fig. 5a). Of the 102 differentially accumulated metabolites, 23 were lipids, fatty acids, or phyto-oxylipins (Table S6). In contrast to monoacylglycerol (MAG) (18:4) isomer 1 that showed increased accumulation, seven glycerolipids, including MAG (18:1) isomer1, MAG (18:3) isomer4, digalactosyl monoacylglycerol (DGMG) (18:2) isomer1, DGMG (18:2) isomer2, DGMG (18:2) isomer3, monogalactosyl monoacylglycerol (MGMG) (18:2) isomer1, and MGMG (18:2) isomer2, were decreased by 3–5 folds when MeJA was applied to the leaves (Fig. 5a; Table S6). The phospholipids LysoPC 18:0 and 18:1 exhibited an over two-fold increase in MeJA-treated leaves (Table S6).

Exogenous MeJA application elevated the levels of JA and JA isoleucine (JA-Ile) by 4.3-fold and 2.8-fold, respectively, in pomegranate leaves (Table S6). Various non-JA phyto-oxylipins, including fatty acid hydroperoxides, epoxy fatty acids, hydroxy fatty acids, keto fatty acids, and acetylenic fatty acids, notably increased in MeJA-treated leaves (Fig. 5a; Table S6). Enhanced accumulation of punicic acid, a PUFA (18:3) that is abundant in pomegranate seed oil, was also observed in leaves treated with MeJA (Table S6).

### Biochemical characterization of a candidate LOX for fatty acid modification in MeJA-treated pomegranate leaves

To explore metabolic genes that are involved in alternation of fatty acid metabolism towards phyto-oxylipins, transcriptomes of mock- and MeJA-treated pomegranate leaves were analyzed for DEGs that are annotated as lipases or LOXs. Two lipase genes *Pgr004895* and *Pgr001125* showed enhanced expression in the 24-h post-MeJA treatment transcriptomes, and were confirmed for significantly increased expression by real-time qPCR analysis (Fig. 5b). On the other hand, the lipase *Pgr024441* showed reduced expression in the transcriptome analysis, which was not supported by the real-time qPCR result (Fig. 5b). Interestingly, elevated expression of *Pgr025417*, a putative LOX, was observed in transcriptomes of leaves collected at 2-h, 6-h, 24-h, and 72-h after MeJA treatment (Fig. S1). Real-time qPCR analysis further indicated a continuously increased expression of *Pgr025417*, ranging from 4- to 20-fold, in leaves collected from 2 to 72-h after MeJA treatment (Fig. 5c).

**Fig. 6:**
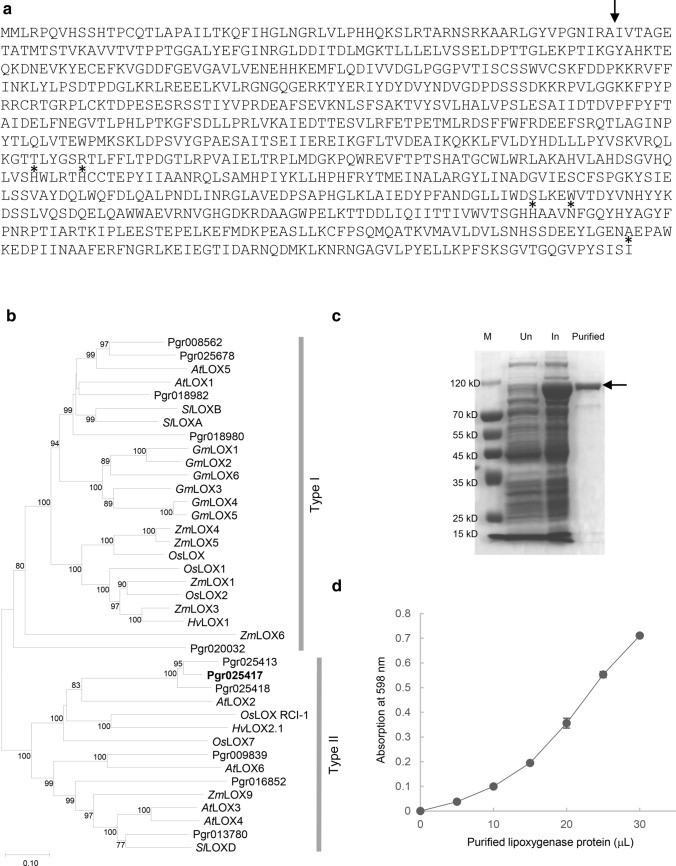
Biochemical characterization of a putative lipoxygenase (LOX) protein. **a** Deduced amino acid sequence of LOX (Pgr025417). The predicted cleavage site of the chloroplast target peptide is indicated with an arrow. Amino acid residues indicated with asterisks correspond to those coordinating the iron atom in the active site of soybean LOX L-1 (Minor et al. 1996). **b** A neighbor-joining phylogenetic tree of 11 LOXs tentatively identified from the pomegranate genome and selected plant LOXs. Bootstrap values (3000 replicates) greater than 60 are shown next to the branches. *At* *Arabidopsis thaliana*, *Gm* *Glycine max*, *Hv* *Hordeum vulgare*, *Os* *Oryza sativa*, *Pgr* *Punica granatum*, *Sl* *Solanum lycopersicum*, *Zm* *Zea mays*. **c** Purification of the recombinant LOX (Pgr025417) protein. The purified protein of the expected size is indicated with an arrow. *M* protein molecular-weight marker, *Un* uninduced, *In* induced. **d** Enzyme activity assays of LOX (Pgr025417) using linoleic acid as substrate. The average and standard deviation of triplicated experiments for each data point are shown

To determine whether the putative LOX (Pgr025417) was involved in the increased phyto-oxylipin production in MeJA-treated leaves, its sequence and activity were examined. When the amino acid sequence of Pgr025417 was analyzed, His, Asn, and Ile residues that correspond to those coordinating the iron atom at the active site of soybean LOX L-1 (Minor et al. 1996) were found in Pgr025417 (Fig. 6a). In addition, Pgr025417 is predicted to be localized to the chloroplast (likelihood probability of 0.9096) with the signal peptide cleaved between 64 and 65 aa (Fig. 6a). Among the 11 putative LOXs (786 aa to 970 aa) identified in the annotated pomegranate genome, 5 other LOXs: Pgr025413, Pgr025418, Pgr009839, Pgr016852, and Pgr013780 are predicted to be chloroplastic besides Pgr025417 and group with type II LOXs characterized in other plants (Fig. 6b). Interestingly, while Pgr008562, Pgr025678, Pgr018982, and Pgr018980 are clustered with dicotyledonous type I LOXs, Pgr020032 is more distantly related to the other type I LOXs (Fig. 6b). To determine the enzymatic activity of Pgr025417, the recombinant protein (906 aa, ~ 102 kDa; Fig. 6c) was assayed using linoleic acid as substrate. Oxidized products of linoleic acid were produced by Pgr025417 as demonstrated by a greater absorption at 598 nm when increased amounts of recombinant protein were present in the reaction mixture (Fig. 6d).

### Transcriptional response to exogenous MeJA application in pomegranate leaves

**Fig. 7:**
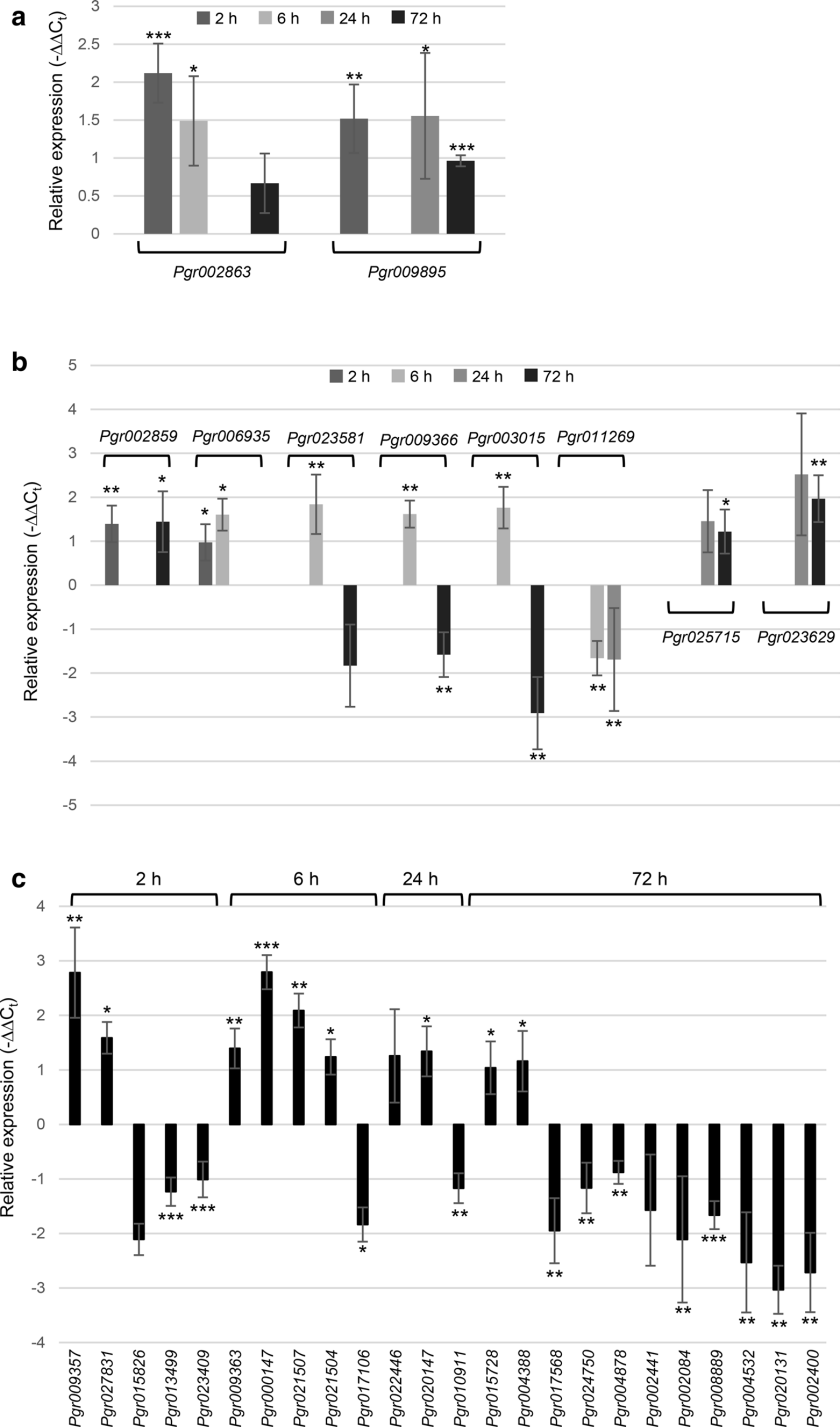
Real-time qPCR analysis of differentially expressed transcription factors identified in the transcriptome analysis. Relative expression (−∆∆C_t_) of transcription factors that demonstrated differential expression in transcriptomes at three **a**, two **b**, and one **c** of the four time points [2-h, 6-h, 24-h, and 72-h after methyl jasmonate (MeJA) application] is shown. Annotation of the transcription factors is listed in Table 1. *P < 0.05; **P < 0.01; ***P < 0.001

To understand whether the changes in HTs, flavonoids, lipids, fatty acids, and phyto-oxylipins are regulated transcriptionally, transcriptomes of MeJA- and mock-treated pomegranate leaves collected at 2-h, 6-h, 24-h, and 72-h were compared and identified 34 transcription factors (TFs) that showed differential expression (|Log_2_FC|> 1, adjusted *P* < 0.05) (Table 1); 31 of these TFs also exhibited significantly changed expression by real-time qPCR analysis (Fig. 7). Notably, a Zinc-finger TF *Pgr009895* showed increased expression at three out of the four time points (2-h, 24-h, and 72-h) (Fig. 7a). The expression of *Pgr002863* (PCF5-like), *Pgr002859* (bZIP1), and *Pgr006935* (auxin response factor) was increased, and *Pgr011269* (MYB) decreased upon MeJA elicitation at two time points (Fig. 7a, b). Both *Pgr009366* (anthocyanin regulatory C1 protein/MYB) and *Pgr003015* (MYB) displayed increased expression at 6-h, but decreased expression at 72-h (Fig. 7b). The expression of 12 TFs was enhanced at only one time point, including *Pgr009357* (2-h), *Pgr027831* (2-h), *Pgr023581* (6-h), *Pgr009363* (6-h), *Pgr000147* (6-h), *Pgr021507* (6-h), *Pgr021504* (6-h), *Pgr020147* (24-h), *Pgr025715* (72-h), *Pgr023629* (72-h), *Pgr015728* (72-h), and *Pgr004388* (72-h) (Table 1; Fig. 7b, c). On the other hand, the expression of 12 TFs was suppressed in MeJA-treated pomegranate leaves at one time point, including *Pgr013499* (2-h), *Pgr023409* (2-h), *Pgr017106* (6-h), *Pgr010911* (24-h), *Pgr017568* (72-h), *Pgr024750* (72-h), *Pgr004878* (72-h), *Pgr002084* (72-h), *Pgr008889* (72-h), *Pgr004532* (72-h), *Pgr020131* (72-h), and *Pgr002400* (72-h) (Table 1; Fig. 7c).

**Table 1:** Transcription factors that show differential expression (|log_2_FC|> 1, adjusted *P* < 0.05) in the transcriptome analysis

Gene name	Annotation	2-h (Log_2_FC)	6-h (Log_2_FC)	24-h (Log_2_FC)	72-h (Log_2_FC)
*Pgr002863*	Transcription factor PCF5	2.406	1.26	–	1.172
*Pgr009895*	Zinc finger protein CONSTANS-LIKE 15	1.49	–	1.201	1.007
*Pgr002859*	Transcription factor VIP1 (bZIP)	1.307	–	–	1.044
*Pgr006935*	Auxin response factor 5	1.047	1.647	–	–
*Pgr023581*	Homeobox-leucine zipper protein ATHB-7	–	2.117	–	– 1.156
*Pgr009366*	Anthocyanin regulatory C1 protein (MYB)	–	1.595	–	– 1.17
*Pgr003015*	Transcription factor MYB44	–	1.44	-	– 1.421
*Pgr011269*	Transcription factor MYB12	–	– 1.779	– 1.984	–
*Pgr025715*	Probable WRKY transcription factor 54	–	–	1.178	1.1
*Pgr023629*	Probable WRKY transcription factor 53	–	–	1.178	1.109
*Pgr009357*	MYB-related protein 66	2.446	–	–	–
*Pgr027831*	Trihelix transcription factor GT-2	1.633	–	–	–
*Pgr015826*	Probable WRKY transcription factor 40	– 2.117	–	–	–
*Pgr013499*	Ethylene-responsive transcription factor ERF008	– 1.379	–	–	–
*Pgr023409*	Transcription activator GLK1	– 1.016	–	–	–
*Pgr009363*	Transcription factor TT2	–	6.379	–	–
*Pgr000147*	Ethylene-responsive transcription factor 9	–	2.556	–	–
*Pgr021507*	Transcription factor MYB23	–	1.908	–	–
*Pgr021504*	Ethylene-responsive transcription factor ERF038	–	1.172	–	–
*Pgr017106*	Transcription factor TGA4	–	– 1.405	–	–
*Pgr022446*	Probable WRKY transcription factor 70	–	–	1.73	–
*Pgr020147*	Probable WRKY transcription factor 65	–	–	1.154	–
*Pgr010911*	Myb-related protein P	–	–	– 1.159	–
*Pgr015728*	Transcription factor HY5 (bZIP)	–	–	–	1.115
*Pgr004388*	MYB-related protein 308	–	–	–	1.032
*Pgr017568*	Transcription factor MYB108	–	–	–	– 1.054
*Pgr024750*	Transcription factor bHLH128	–	–	–	– 1.056
*Pgr004878*	Ethylene-responsive transcription factor RAP2-12	–	–	–	–1.062
*Pgr002441*	NAC domain-containing protein 72	–	–	–	– 1.139
*Pgr002084*	Transcription activator GRF3	–	–	–	– 1.394
*Pgr008889*	MYB-related protein 306	–	–	–	– 1.407
*Pgr004532*	NAC domain-containing protein 100	–	–	–	–1.459
*Pgr020131*	Transcription factor MYB108	–	–	–	– 1.514
*Pgr002400*	Homeobox-leucine zipper protein ATHB-52	–	–	–	– 1.577

*FC* fold change

To assess the transcriptional regulation of candidate genes that may function in modulating flavonoid and fatty acid/phyto-oxylipin pathways upon MeJA induction, TF-binding sites in the promoter regions of the candidate genes were predicted using PlantRegMap (Tian et al. 2020) with TFs bioinformatically identified from *Eucalyptus grandis*, which is closely related to pomegranate in Myrtales (Table S7). For *Pgr025417* (putative *LOX*), 80 binding sites of 65 TFs were identified, including abundant binding sites for MYB (10 TFs, 15 sites) and WRKY (11 TFs, 18 sites), but without binding sites for bZIP or zinc finger TFs (Table S7). Because CHS and CHI are positioned at the entry point of flavonoid and anthocyanin biosynthetic pathways and showed reduced gene expression in transcriptome and real-time qPCR analyses (Figs. S1 and 4), the promoters of putative *CHS* (*Pgr005566*) and *CHI* (*Pgr025966*) were also analyzed for putative TF-binding sites. For *CHS*, there are 104 binding sites of 76 TFs, with MYB (20 TFs, 23 sites) and bHLH (13 TFs, 23 sites) being the most abundant TFs (Table S7). For *CHI*, there are 93 binding sites of 78 TFs, with bHLH (16 TFs, 23 sites) and WRKY (13 TFs, 13 sites) being the most abundant TFs (Table S7).

## Discussion

Unique metabolic changes induced by exogenous MeJA application suggests functions of HTs, methylated flavones/flavonols, and phyto-oxylipins in pomegranate response to MeJA.

MeJA has a demonstrated role in eliciting stress response in plants (Cheong and Choi 2003). The accumulation of two HT pathway intermediates β-glucogallin and pentagalloylglucose in leaves was induced at 30-h after MeJA application (Fig. 2b), suggesting that they act in response to abiotic and biotic stresses in the environment. This result also corroborates the function of HTs in protecting pomegranate from abiotic stresses in the tissue of fruit peels (Schwartz et al. 2009; Habashi et al. 2019). Corresponding to the rise in HT metabolite accumulation, shikimate and HT biosynthetic pathway genes also showed increased expression (Fig. 1). Previous biochemical characterization of four grapevine (*Vitis vinifera*) SDH isoforms indicated that only *Vv*SDH3 and *Vv*SDH4 could produce gallic acid from 3-dehydroshikimate (Bontpart et al. 2016). According to our transcriptome and real-time qPCR analyses, *PgSDH3_1* and *PgSDH3_2*, homologs of *VvSDH3*, but not *PgSDH4*, a homolog of *VvSDH4*, exhibited induced expression by MeJA (Figs. S1 and 1). This observation suggests that *Pg*SDH3_1 and *Pg*SDH3_2 are likely involved in the synthesis of HTs in response to MeJA treatment and environmental stresses. Additionally, enhanced expression of *PgUGT8423* and *PgUGT84A24*, which encode enzymes catalyzing the committed step of HT biosynthesis, by MeJA application further supports the role of HTs in stress response.

At 72-h after MeJA induction, an overall suppression of flavonoids and anthocyanins was found in pomegranate leaves (Fig. 3; Table S6), which is contrary to the increased accumulation of these compounds observed in many other plants (Pandey et al. 2016; De Geyter et al. 2012; Shafiq et al. 2011; Flores and Ruiz del Castillo 2014; Portu et al. 2015). However, three methylated flavones and flavonols: di-*O*-methylquercetin, selgin 5-*O*-hexoside, and chrysoeriol *O*-hexosyl-*O*-hexoside accumulated at a greater level despite the reduction of non-methylated biosynthetic precursors (Fig. 3; Table S6). Mono- and di-*O*-methylated quercetin are secreted from trichomes of solanaceous species and have been proposed to act in plant defense, possibly in a species-specific manner (Wollenweber and Dörr 1995; Roda et al. 2003). It was also shown that glycosides of chrysoeriol, luteolin, and apigenin deterred feeding of aquatic herbivores on *Potamogeton lucens* (pondweed) leaves in in vitro assays (Erhard et al. 2007). The induced accumulation of methylated flavones/flavonols in pomegranate leaves suggests that they may play a role in MeJA-elicited stress response, and have implications for defense against wounding, pathogens, and herbivores in this tree species.

Besides HTs, flavonoids, and anthocyanins, additional notable metabolic changes included the mobilization of free fatty acids from lipids and the biosynthesis of phyto-oxylipins at 72-h after MeJA application (Fig. 5a). The threefold increase in punicic acid (18:3, *cis*-9, *trans*-11, *cis*-13) upon MeJA induction suggests a potential role of this PUFA with a three conjugated double bond system in stress signaling or direct chemical defense of pomegranate plants (Table S6). The concurrent reduction of MAG, MGMG, and DGMG also suggests that punicic acid could be usually conjugated to these glycerolipids in pomegranate leaves (Fig. 5a; Table S6). Of the phyto-oxylipins, the rise in JA and JA-Ile in MeJA-treated pomegranate leaves could be due to demethylation of the exogenously applied MeJA to form JA, which is subsequently converted to JA-Ile (Table S6) (Stuhlfelder et al. 2004). It may also suggest that MeJA can directly regulate the biosynthesis of JA and its derivatives in pomegranate. Volatile compounds derived from phyto-oxylipins are reportedly involved in signaling response to wounding and pathogen attacks (Lim et al. 2017). Recent studies have also revealed direct antimicrobial roles of non-JA phyto-oxylipins, though the mechanistic basis for such functions is still unclear (Deboever et al. 2020). The inducible production of phyto-oxylipins in pomegranate corroborates the previous observations in other plants. It remains to be determined whether these phyto-oxylipins are biocidal or function to stimulate innate immune response in pomegranate.

### The modified flavonoid and anthocyanin, but not phyto-oxylipin, metabolism upon MeJA induction is at least partially regulated at the transcriptional level

Consistent with the generally decreased accumulation of flavonoids and anthocyanins, genes encoding CHS and CHI, two enzymes positioned at the entry point of flavonoid and anthocyanin biosynthesis, showed reduced expression in MeJA-treated leaves (Fig. 4). Intriguingly, a pomegranate homolog (Pgr009366) of the maize MYB TF anthocyanin regulatory protein C1 was initially upregulated in MeJA-treated leaves at 6-h, but then downregulated in MeJA-treated leaves at 72-h (Table 1; Fig. 7b). A pomegranate homolog (Pgr010911) of the maize MYB TF P was downregulated in MeJA-treated leaves at 24-h (Table 1; Fig. 7c). As demonstrated in studies in maize, C1 activates several genes in flavonoid and anthocyanin biosynthesis, such as *CHS* (*C2*), *dihydroflavonol reductase* (*A1*), *anthocyanidin 3-O-glucosyltransferase* (*BZ1*), and *leucoanthocyanidin dioxygenase* (*A2*) (Lesnick and Chandler 1998; Sainz et al. 1997; Quattrocchio et al. 1993). P, on the other hand, controls the expression of *A1*, but not *BZ1* (Grotewold et al. 1994). In maize, C1 (MYB TF) works in concert with R (bHLH TF) to regulate the expression of anthocyanin biosynthetic genes (Mol et al. 1998; Goff et al. 1992). *Pgr024750*, a bHLH TF, was also downregulated in MeJA-treated leaves at 72-h and could a potential partner of the pomegranate C1 homolog (Table 1; Fig. 7c). Furthermore, binding sites for MYB and bHLH TFs were identified in the promoter regions of putative pomegranate *CHS* and *CHI* through bioinformatics analysis (Table S7). Taken together, these results suggest that the overall decreases in flavonoids and anthocyanins is at least partially attained through transcriptional control of the early-step biosynthetic genes.

A reduction of several glycerolipids was observed in MeJA-treated leaves (Fig. 5). However, pomegranate homologs (GenBank accession numbers: XP_031374952 and XP_031374953) of the Arabidopsis Wrinkled1 (*At*WRI1), an APETALA2 (AP2) family TF that is considered a “master” regulator for plant lipid biosynthesis (Cernac and Benning 2004), expressed similarly in mock- and MeJA-treated leaves (data not shown). Functional characterization of the TFs responsive to MeJA induction (Table 1; Fig. 7) could potentially reveal a regulatory role for lipid metabolism in pomegranate leaves.

The pomegranate LOX (Pgr025417) with continuously increased expression after exogenous MeJA application, catalyzed fatty acid oxidation and was predicted to localize to the chloroplast (Figs. 5c and 6). Therefore, Pgr025417 is likely involved in JA biosynthesis that takes place in this subcellular compartment (Fig. 6). Of the 11 putative LOXs in pomegranate, 5 are cytosolic (type I) and 6 are chloroplastic (type II) (Fig. 6b). Since non-JA phyto-oxylipins are synthesized in the cytosol (Ponce de León et al. 2015), one or more of the type I LOXs may be responsible for converting PUFA to non-JA phyto-oxylipins upon MeJA induction. Considering that the LOX (Pgr025417) with a sustained increase in gene expression in MeJA-treated leaves is located in a subcellular compartment different from non-JA phyto-oxylipins, the enzymes responsible for producing MeJA-induced non-JA phyto-oxylipins are likely not modulated transcriptionally.

### Exogenous MeJA application induced transcriptional responses in pomegranate leaves

A recent study in Arabidopsis revealed that a complex regulatory network enriched with MYC (bHLH), ethylene response factor (ERF), and MYB family TFs was involved in the early MeJA-induced transcriptional response (Hickman et al. 2017). Pomegranate homologs of ERF TFs, including *Pgr013499* (2-h), *Pgr000147* (6-h), and *Pgr021504* (6-h), and homologs of MYB TFs, including *Pgr009357* (2-h), *Pgr009366* (6-h), *Pgr003015* (6-h), and *Pgr011269* (6-h), were among the TFs with varied expression in MeJA-treated leaves collected at early time points (Table 1; Fig. 7). As discussed above, pomegranate homologs of the maize flavonoid and anthocyanin regulatory proteins C1 and P (MYB TFs), and a bHLH TF showed decreased expression and could potentially control the overall reduction of flavonoids and anthocyanins. In addition, the promoter region of *LOX* (*Pgr025417*) contains binding sites of WRKY, MYB, and bZIP TFs (Table S7), which suggests that they could be regulated by the differentially expressed TFs belonging to these TF families in response to MeJA application.

### Comparative gene expression and metabolite analysis of MeJA-treated leaves facilitates the interrogation of diverse phenolic pathways in fruits

Understanding metabolic and transcriptional responses of pomegranate leaves to MeJA application is clearly pertinent to improving plant health and productivity (i.e. fruit production). In addition, the comparative gene expression and metabolite analysis of MeJA-treated leaves also provides an opportunity to elucidate the regulation of HT, flavonoid, and anthocyanin pathways that are present in both leaf and fruit tissues (Bar-Ya'akov et al. 2019). The phenolic compounds HTs, flavonoids, and anthocyanins contribute greatly to the human health-beneficial activities of pomegranate fruits, yet the control of their production and accumulation has not been extensively explored in pomegranate (for flavonoids and anthocyanins) or any plant species (for HTs). For example, previous studies on MeJA-applied pomegranate fruits determined the levels of anthocyanins and flavonoids in fruits after harvest, without investigating the expression of structural or regulatory genes related to these metabolites (Koushesh Saba and Zarei 2019; García-Pastor et al. 2020). On the other hand, the current study uncovered TFs (*e.g.* MYB TFs) that showed differential expression in MeJA- and mock-treated leaves with expression patterns similar to those of HT, flavonoid, and anthocyanin biosynthetic genes (Fig. 7; Table 1). These TFs are potentially involved in the regulation of HT, flavonoid, and anthocyanin pathways thus warrant further investigation in the future.

It is worth noting that our study revealed a specific increase in methylated flavonoids and general decreases in other flavonoids in pomegranate leaves treated with MeJA, suggesting a potential role of methylated flavonoids in defending pomegranate leaves against pathogens (Fig. 3; Table S6). The previous report on pomegranate fruits treated with MeJA showed enhanced flavonoid accumulation, though only the total flavonoid content was determined without quantifying individual flavonoid molecules (Koushesh Saba and Zarei 2019). As such, a more in-depth analysis of pomegranate fruits treated with MeJA is needed to allow for a side-by-side comparison of flavonoid changes in leaf and fruit tissues. Contrasting to increased anthocyanins in pomegranate fruits after MeJA applications (García-Pastor et al. 2020), the level of anthocyanins was reduced in MeJA-treated leaves (Fig. 3; Table S6). This disparity could be due to distinct roles that anthocyanins play in leaf and fruit tissues upon pathogen attacks. In fact, the differential accumulation of anthocyanins in leaves and fruits underscores the need for examining MeJA responses in different pomegranate tissues.

## Concluding remarks

Our study revealed unique metabolic changes in HTs, flavonoids, anthocyanins, and phyto-oxylipins in pomegranate leaves triggered by exogenous MeJA application. Transcriptome and biochemical analyses suggested that, while suppression of multiple flavonoids and anthocyanins occurs at least partially at the transcriptional level, increased biosynthesis of non-JA phyto-oxylipins is likely not controlled transcriptionally. This work instigates further investigations on the regulatory architecture of HT, flavonoid, and anthocyanin metabolism, the orchestration of metabolic responses in pomegranate leaves to MeJA application, as well as the role that metabolites with induced accumulation play in signaling and/or direct chemical defense.

### *Author contributions statement*

LC and LT conceived the study. LC and WS performed experiments. LC, WS, and LT analyzed data. LC, WS, and LT wrote the manuscript. All authors read and approved the final manuscript.

## Supplementary Information

Below is the link to the electronic supplementary material.

Supplementary file1 (PDF 170 KB ) Fig. S1 KEGG pathway enrichment analysis. The enrichment score [-log10(Padjust); primary Y axis] and the number of differentially expressed genes (secondary Y axis) at a 2-h, b 6-h, c 24-h, and d 72-h, after methyl jasmonate (MeJA) application relative to mock-application controls are shown. KEGG, Kyoto Encyclopedia of Genes and Genomes

Supplementary file2 (PDF 145 KB)

Supplementary file3 (PDF 90 KB)

Supplementary file4 (PDF 90 KB)

Supplementary file5 (PDF 72 KB)

Supplementary file6 (XLSX 90 KB)

Supplementary file7 (XLSX 35 KB)

Supplementary file8 (XLSX 36 KB)

## Data Availability

The transcriptome datasets generated and analyzed during the current study are available in the Sequence Read Archive (SRA) at NCBI under the accession number PRJNA600139. The metabolite profiling datasets generated and analyzed during the current study are included in this published article and its supplementary files.

## Abbreviations

AP2: APETALA2
CE: Collision energy
CHI: Chalcone isomerase
CHS: Chalcone synthase
∆∆C_t_: Comparative C_t_
DEG: Differentially expressed gene
DGMG: Digalactosyl monoacylglycerol
DMAB: 3-(Dimethylamino)benzoic acid
α-DOX: α-Dioxygenase
DP: Declustering potential
HPLC: High-performance liquid chromatography
HPO: Hydroperoxides
HT: Hydrolyzable tannin
IPTG: Isopropyl-β-D-thiogalactoside
JA: Jasmonic acid
JA-Ile: JA isoleucine
LB: Luria Bertani
LC–ESI–MS/MS: Liquid chromatography electrospray ionization tandem mass spectrometry
LIT: Linear ion trap
LOX: Lipoxygenase
MAG: Monoacylglycerol
MAPK: Mitogen-activated protein kinase
MBTH: 3-Methyl-2-benzothiazolinone
MEGA: Molecular evolutionary genetics analysis
MeJA: Methyl jasmonate
MGMG: Monogalactosyl monoacylglycerol
MRM: Multiple reaction monitoring
MS2T: MS/MS spectral tag
MUSCLE: Multiple sequence comparison by log-expectation
OMT: *O*-Methyltransferase
OPLS-DA: Orthogonal partial least squares discriminant analysis
PUFA: Polyunsaturated fatty acid
PXG: Peroxygenase
qPCR: Quantitative PCR
QQQ: Triple quadrupole
RSEM: RNA-Seq by expectation maximization
RT: Reverse transcription
SAM: *S*-Adenosyl-methionine
TF: Transcription factors
TFA: Trifluoroacetic acid
TPM: Transcripts per kilobase million
VIP: Variable importance in projection
WRI1: Wrinkled1
