# Supplementary figures and images for: Methyl jasmonate elicits distinctive hydrolyzable tannin, flavonoid, and phyto-oxylipin responses in pomegranate (Punica granatum L.) leaves
Source: Planta. 2021 Sep 29;254(5):89. doi: 10.1007/s00425-021-03735-9 (PMC8481150; doi:10.1007/s00425-021-03735-9)

**a**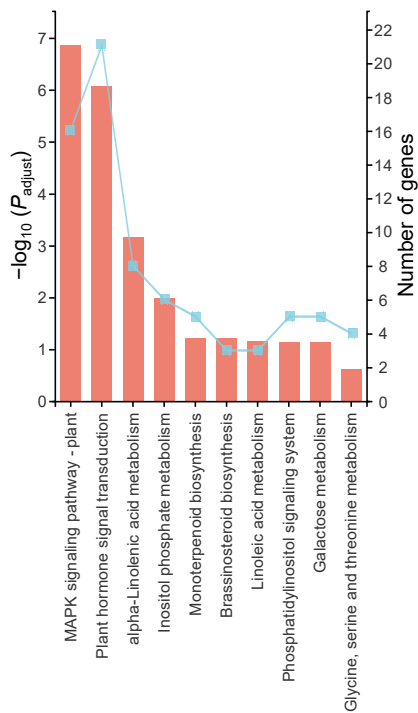**b**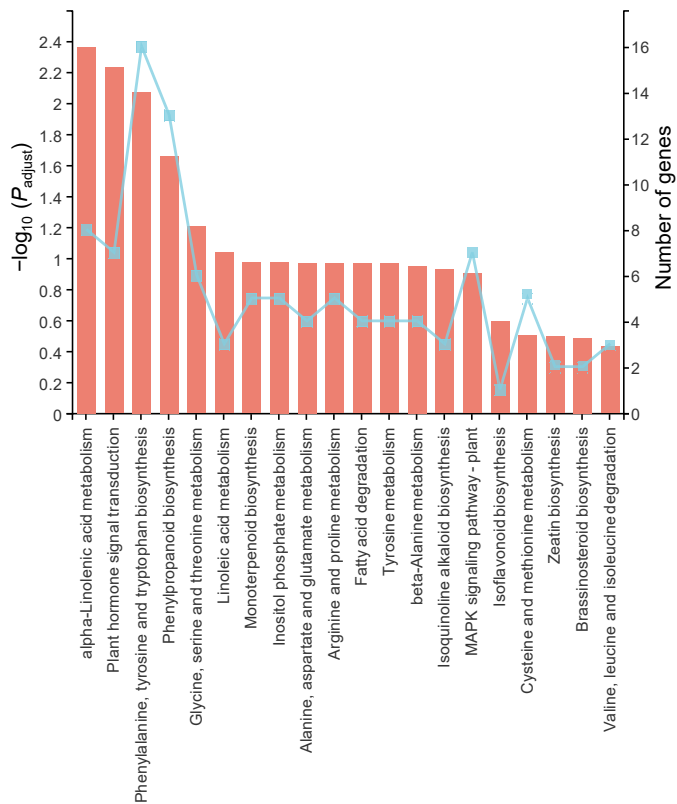**c**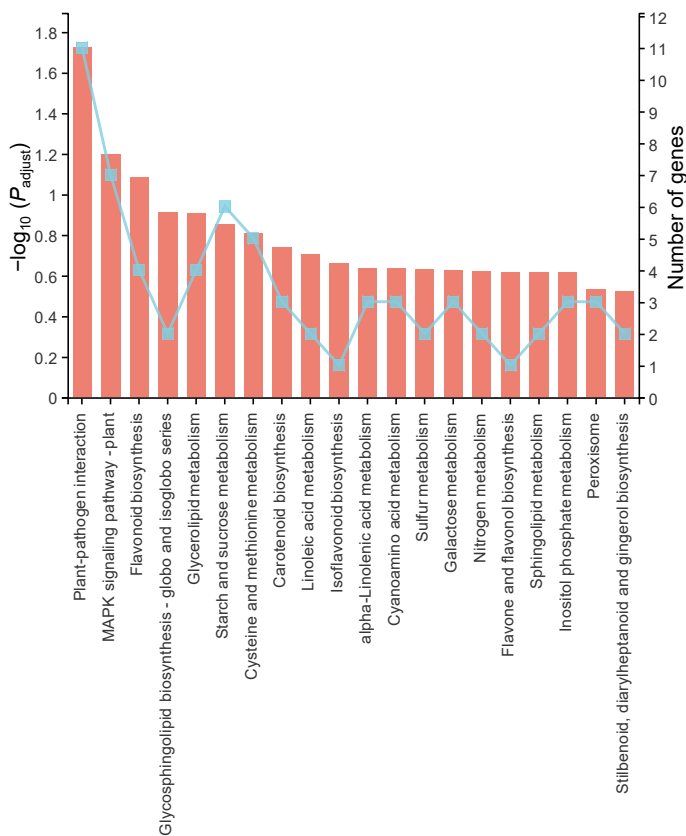**d**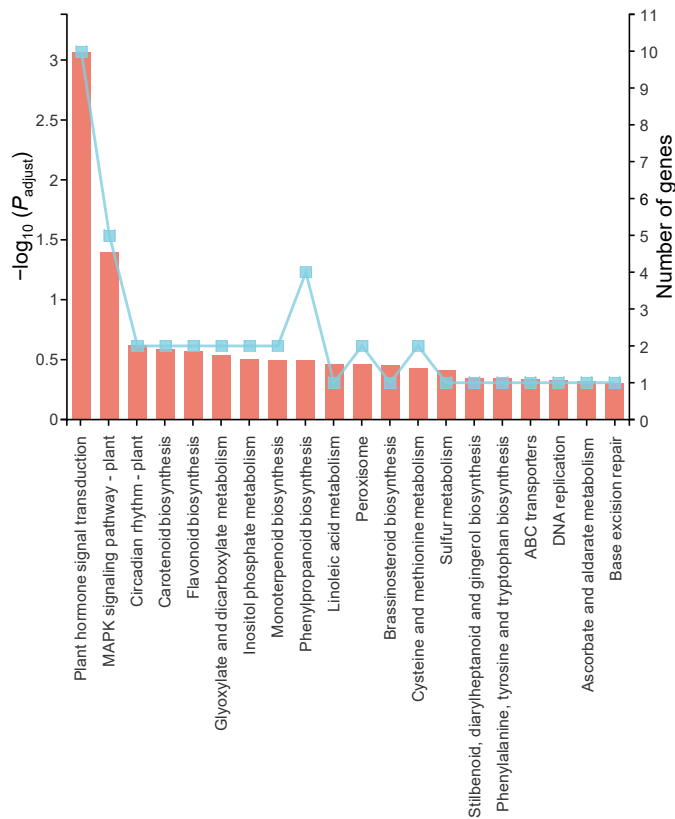

Supplement: Supplementary file 1 — Supplementary file1 (PDF 170 KB ) Fig. S1 KEGG pathway enrichment analysis. The enrichment score [-log10(Padjust); primary Y axis] and the number of differentially expressed genes (secondary Y axis) at a 2-h, b 6-h, c 24-h, and d 72-h, after methyl jasmonate (MeJA) application relative to mock-application controls are shown. KEGG, Kyoto Encyclopedia of Genes and Genomes [file 425_2021_3735_MOESM1_ESM.pdf]
