# Supplementary material for: Methyl jasmonate elicits distinctive hydrolyzable tannin, flavonoid, and phyto-oxylipin responses in pomegranate (Punica granatum L.) leaves
Source: Planta. 2021 Sep 29;254(5):89. doi: 10.1007/s00425-021-03735-9 (PMC8481150; doi:10.1007/s00425-021-03735-9)
Supplement: Supplementary file 2 — Supplementary file2 (PDF 145 KB) [file 425_2021_3735_MOESM2_ESM.pdf]

Table S1. Primers used for real-time qPCR analysis.

Gene	Forward (5'→3')	Reverse (5'→3')	Amplicon size (bp)	Amplification efficiency
Pgr002863	CAGCCACTTGATTCTGTGAGCA	AAGAGTGAAGAGACAAGCCCAGAT	76	94.8%
Pgr009895	TTGGTGCTTTGTCACGAGTGT	ATCGAGTCCTGAAACCCATAGA	215	89.3%
Pgr002859	CGGCTCTGGCTCAGGATAAG	GAGATGGGTGGCTGAGATGA	151	107.9%
Pgr006935	CGACACCTTCTTACGACTGGCT	TTTGCTCGCCTCACGCCTAC	122	106.2%
Pgr023581	CACGACTACGCCATCCTCAG	GGCTCCGTCTTGGTTTCAT	191	109.9%
Pgr009366	CGATACCCGTGGATAAAGCA	GTCAAGCAGAAATGCCAAAGAT	101	100.6%
Pgr003015	CCCCAGAACTGGAACTTGAT	GTGAACGCCTTCTTGTTGATT	110	97.8%
Pgr011269	TAATCCTAAACAACCCAAAGCCC	TCGTCCTCATCCCCTCCAGTC	105	99.3%
Pgr025715	TTCTGCCACTACAGGAGACGAT	CTGCTGGTTGTCTTCCATTCTC	157	103.7%
Pgr023629	TCCGAATGTATTTGAGGTTGTG	ACCCGTTATCTTGTTCCTGTCT	119	91.2%
Pgr009357	TGGCAAGCAATCAGAGTATCGTA	CTGCTGTGAGGCTGTGGTCTAC	122	99.2%
Pgr027831	TGATGGTGAGACCTGAACGACA	GGTCTCAATGCTATTCTCCCGTT	83	97.3%
Pgr015826	AATCGGAAAACAAGAAACTCACG	TCACATTATGCCATTCGTAGTCTTC	141	95.0%
Pgr013499	AACAGGGGTCTGAACGGCAT	GTCAGAGTAATCCTCAGGGTTGG	153	89.3%
Pgr023409	ACAATCCTCAAGGGAAGCGAAA	CGATGGGACCGATATTTCTGG	187	88.6%
Pgr009363	GTAGAAGTGCGAAGAGTAGCCG	AAAGACCACCTGTTGCCTAAGAG	133	96.8%
Pgr000147	AAACATGAACGAGCAGCTATACCA	CAGATTGACCACTGACGACGAGT	109	92.5%
Pgr021507	CAGGCAGGACTGATAACGAGATT	CCTGAGAGTTTTCGGGAGTTTTA	141	94.7%
Pgr021504	AGAGGAAGATTACGGCGACCC	CAGTCCCAGTAAAACGGGTCAT	162	95.6%
Pgr017106	TGGGTAGATGAACAGAATAGGCA	CGTTCTCTACGAGCACCCGA	94	98.8%
Pgr022446	GCTTCAACATCGTCTTCCCG	GAAATCCCCCCCTGACTAAGAG	258	99.2%
Pgr020147	TCGGTCCTCGTCGTCACTTACAC	TCGTGTCCAGTTGGCACAGTGT	256	94.4%
Pgr010911	GAGAACGGAACGGGTGGTAG	CCCTCAAATGGTCCTAAAACTTCA	98	94.4%
Pgr015728	GGAGGAGCCCTGCTGATAAA	GTCCTTCACCCTCACCTCCA	125	93.5%
Pgr004388	ATTGCCTACATCCGTACCCAC	TTATCTCATTGTCCGTCCTTCC	238	107.8%
Pgr017568	TCTATTAGCCAAATGTGCCG	TAATCTCATTGTCTGTCCTCCC	200	88.6%
Pgr024750	CTTATGCCACTACAAGTTTCGG	CATCTCAAGAGTTGTCTGTGGC	161	109.8%
Pgr004878	GGTCTGGCTCGGAACCTTTA	GGCTTCGGTGGGTTTGACTT	156	93.7%
Pgr002441	CAACAACCGCACGAACAGA	TGGCATAATTGGCAACCTGA	172	106.9%
Pgr002084	TCCACAACCTGCTTTCTATTTTCAT	AGATTAAAATAACCGGAGATGCG	129	89.2%
Pgr008889	GCTCAGCCCAAACCAAATCA	CAAACCCGCCATCACTCG	120	91.4%
Pgr004532	GGACGACGAGCCCATCAA	CGCCCTGTTCGTCCTCAA	247	114.5%
Pgr020131	GCTTGACTGTTCGGGTGA	TTAATCTAAGGGAGTGCTATGC	211	99.8%
Pgr002400	GCGGTGGGAGTTCTTCAAAT	GGACCATTAGCGAACATCACG	154	91.2%
Pgr001759 (PgDAHPS)	CTGCCAGTCCTCTGCTACCCTTA	GTCTCCCTGCTCGCTGTGCT	118	95.1%
Pgr013674 (PgDHQS)	TAGGGAACTGTGTCTTCACTGGG	CGAACTACAACTAAACACTTGGCAC	141	96.1%
Pgr020271 (PgSDH3_1)	AAAGAACTGGCTGATAAGGTAGGA	CCCATCGGCATTTGGTTTCA	126	93.3%
Pgr019030 (PgSDH3_2)	TGAGATTGACCCGATTGCTAAGA	AGCAGTAATGGCACCGATGTAAT	109	92.0%
Pgr019029 (PgSDH_4)	AACAAATCGCTGGTTCTCAATAG	CCTGCCATTCTTATCGTCATTGT	147	92.2%
Pgr003363 (PgUGT84A23)	GTGGCTGGACTCGAAACCA	GAAGCCCATACGCGATCTCA	101	94.2%
Pgr028532 (PgUGT84A24)	GCCGAGAACAAGCTGATCATG	TCCACTTGAGCGCATTCTCC	103	96.5%
Pgr005566	GGACGCTAGGCAGGACATT	GATCTTCGACTTGGGCTGAC	100	91.0%
Pgr025966	CCCACCTGGTTCCTCAATCCT	TTCCGTCTTCTGGGATCTTCC	92	104.5%
Pgr025417	TGTGAGCTTAATGTATTTGTGCGG	TTCCATGAAAAGTATGATGCGCGT	113	103.8%
Pgr025413	CAAGGCGGATGAGATGGGATA	TGTTAGAGCCGCCCTAGTACATCA	80	96.7%
Pgr004895	TACTCGCCGAAAATAGTCCCAA	CTTCTTATGACCCTCCTGCTGCT	101	99.8%
Pgr001125	AACATACTGGGGGATGAGGACAC	GGTAGTCGTTGCTTCCCATTCC	82	101.6%
Pgr024441	GTGCCAAAGGTGGAGTATTTCAGA	GGAAATAGCCAGCAGTCAGGTCAT	108	99%
