# Supplementary material for: Methyl jasmonate elicits distinctive hydrolyzable tannin, flavonoid, and phyto-oxylipin responses in pomegranate (Punica granatum L.) leaves
Source: Planta. 2021 Sep 29;254(5):89. doi: 10.1007/s00425-021-03735-9 (PMC8481150; doi:10.1007/s00425-021-03735-9)
Supplement: Supplementary file 3 — Supplementary file3 (PDF 90 KB) [file 425_2021_3735_MOESM3_ESM.pdf]

Table S2. Summary of RNAseq data. H, mock treatment; M, methyl jasmonate treatment.

Sample	Raw reads	Raw bases (bp)	Clean reads	Clean bases (bp)	Error rate (%)	Q20 (%)	Q30 (%)	GC content (%)
H2_1	57,633,888	8,702,717,088	57,080,872	8,445,758,248	0.0242	98.4	94.95	52
H2_2	50,067,808	7,560,239,008	49,570,690	7,400,638,397	0.0246	98.21	94.48	52.12
H2_3	51,743,812	7,813,315,612	51,245,804	7,629,167,911	0.0242	98.37	94.86	51.9
H6_1	54,807,280	8,275,899,280	54,270,446	8,051,472,506	0.0242	98.38	94.87	51.79
H6_2	54,000,878	8,154,132,578	53,494,292	7,936,572,608	0.0243	98.36	94.83	51.67
H6_3	60,493,680	9,134,545,680	59,842,060	8,831,744,887	0.0244	98.29	94.68	51.96
H24_1	54,708,448	8,260,975,648	54,186,356	8,025,676,382	0.0242	98.38	94.89	51.97
H24_2	51,962,812	7,846,384,612	51,457,692	7,655,388,803	0.0243	98.36	94.81	52.05
H24_3	52,901,486	7,988,124,386	52,372,814	7,789,353,546	0.0242	98.37	94.86	52.32
H72_1	59,894,336	8,984,150,400	56,851,836	8,207,099,492	0.0153	97.01	91.84	50.27
H72_2	57,347,004	8,602,050,600	54,658,114	7,905,074,524	0.0151	97.09	92.03	50.24
H72_3	51,181,158	7,677,173,700	48,435,374	6,989,405,884	0.0155	96.92	91.6	50.73
M2_1	51,229,648	7,735,676,848	50,727,230	7,544,290,673	0.0243	98.34	94.81	52.03
M2_2	57,894,616	8,742,087,016	57,300,352	8,505,367,598	0.0241	98.41	94.99	51.83
M2_3	57,457,538	8,676,088,238	56,934,946	8,456,239,946	0.0242	98.39	94.92	51.9
M6_1	58,539,842	8,839,516,142	57,981,944	8,594,202,972	0.0243	98.36	94.85	51.36
M6_2	52,019,170	7,854,894,670	51,479,888	7,632,211,613	0.0242	98.38	94.88	51.36
M6_3	56,450,284	8,523,992,884	55,888,142	8,276,094,868	0.0243	98.36	94.84	51.45
M24_1	54,251,554	8,191,984,654	53,719,216	7,978,482,498	0.0242	98.37	94.87	52.11
M24_2	51,600,308	7,791,646,508	51,056,638	7,576,246,179	0.0243	98.33	94.76	51.73
M24_3	54,392,858	8,213,321,558	53,808,112	7,970,828,582	0.0244	98.32	94.72	51.33
M72_1	53,529,336	8,029,400,400	50,930,302	7,357,914,264	0.0152	97.05	91.91	50.6
M72_2	57,907,452	8,686,117,800	54,967,572	7,944,470,328	0.0154	96.99	91.77	50.89
M72_3	55,564,242	8,334,636,300	52,451,730	7,582,158,338	0.0144	97.35	92.7	51
