# Supplementary material for: Methyl jasmonate elicits distinctive hydrolyzable tannin, flavonoid, and phyto-oxylipin responses in pomegranate (Punica granatum L.) leaves
Source: Planta. 2021 Sep 29;254(5):89. doi: 10.1007/s00425-021-03735-9 (PMC8481150; doi:10.1007/s00425-021-03735-9)
Supplement: Supplementary file 4 — Supplementary file4 (PDF 90 KB) [file 425_2021_3735_MOESM4_ESM.pdf]

Table S3. RNAseq mapped reads and distributions.

Sample	Total mapped	Multiple mapped	Uniquely mapped	Introns	3'UTR	CDS	5'UTR	Intergenic
H2_1	55022608(96.39%)	1758901(3.08%)	53263707(93.31%)	3413403.0(4.6%)	2022933.0(2.72%)	62392266.0(84.02%)	1611715.0(2.17%)	4816926.0(6.49%)
H2_2	47808043(96.44%)	1496423(3.02%)	46311620(93.43%)	2994339.0(4.6%)	1809974.0(2.78%)	54670749.0(84.06%)	1348822.0(2.07%)	4211875.0(6.48%)
H2_3	49464134(96.52%)	1401485(2.73%)	48062649(93.79%)	3133952.0(4.63%)	1805050.0(2.66%)	57318708.0(84.61%)	1379636.0(2.04%)	4106327.0(6.06%)
H6_1	52337990(96.44%)	1568523(2.89%)	50769467(93.55%)	3080362.0(4.27%)	1923684.0(2.66%)	61268025.0(84.83%)	1469013.0(2.03%)	4479460.0(6.2%)
H6_2	51543334(96.35%)	1514420(2.83%)	50028914(93.52%)	3036622.0(4.28%)	1863096.0(2.62%)	60260808.0(84.86%)	1467006.0(2.07%)	4383199.0(6.17%)
H6_3	57781307(96.56%)	1689797(2.82%)	56091510(93.73%)	3310619.0(4.18%)	2124280.0(2.68%)	67317678.0(84.96%)	1640344.0(2.07%)	4841942.0(6.11%)
H72_1	55365832(97.39%)	1291195(2.27%)	54074637(95.12%)	4200288.0(5.19%)	2185572.0(2.7%)	62476085.0(77.23%)	1652884.0(2.04%)	10382581.0(12.83%)
H72_2	53252114(97.43%)	1254411(2.3%)	51997703(95.13%)	4069262.0(5.24%)	2146592.0(2.76%)	59733576.0(76.89%)	1552455.0(2.0%)	10182827.0(13.11%)
H72_3	47112029(97.27%)	1065995(2.2%)	46046034(95.07%)	3453847.0(5.06%)	1849472.0(2.71%)	52903240.0(77.45%)	1416014.0(2.07%)	8687320.0(12.72%)
H24_1	52345749(96.6%)	1415690(2.61%)	50930059(93.99%)	3512121.0(4.88%)	1991227.0(2.77%)	60694449.0(84.35%)	1509969.0(2.1%)	4247420.0(5.9%)
H24_2	49712776(96.61%)	1409367(2.74%)	48303409(93.87%)	3347696.0(4.92%)	1831811.0(2.69%)	57415246.0(84.33%)	1477705.0(2.17%)	4012009.0(5.89%)
H24_3	50623648(96.66%)	1345335(2.57%)	49278313(94.09%)	3407692.0(4.9%)	1845918.0(2.66%)	58934272.0(84.81%)	1393319.0(2.01%)	3906218.0(5.62%)
M2_1	48836568(96.27%)	1364995(2.69%)	47471573(93.58%)	2998325.0(4.49%)	1739241.0(2.6%)	56684999.0(84.84%)	1374957.0(2.06%)	4013016.0(6.01%)
M2_2	55181297(96.3%)	1631181(2.85%)	53550116(93.46%)	3449975.0(4.58%)	1989553.0(2.64%)	63663117.0(84.49%)	1630223.0(2.16%)	4614751.0(6.12%)
M2_3	54784641(96.22%)	1545011(2.71%)	53239630(93.51%)	3406964.0(4.55%)	1990425.0(2.66%)	63533218.0(84.77%)	1571704.0(2.1%)	4442044.0(5.93%)
M6_1	55664804(96.0%)	1713217(2.95%)	53951587(93.05%)	3320018.0(4.34%)	2025565.0(2.65%)	64480992.0(84.33%)	1738038.0(2.27%)	4895167.0(6.4%)
M6_2	49618966(96.39%)	1503295(2.92%)	48115671(93.46%)	3008827.0(4.4%)	1811950.0(2.65%)	57627065.0(84.25%)	1609507.0(2.35%)	4343010.0(6.35%)
M6_3	53808756(96.28%)	1644966(2.94%)	52163790(93.34%)	3244519.0(4.36%)	1936537.0(2.6%)	62884648.0(84.49%)	1732287.0(2.33%)	4629718.0(6.22%)
M24_1	51722390(96.28%)	1481319(2.76%)	50241071(93.53%)	3170446.0(4.49%)	1942970.0(2.75%)	59664853.0(84.5%)	1522730.0(2.16%)	4304740.0(6.1%)
M24_2	49232115(96.43%)	1483171(2.9%)	47748944(93.52%)	3140861.0(4.69%)	1840345.0(2.75%)	56265370.0(84.0%)	1449386.0(2.16%)	4288830.0(6.4%)
M24_3	51813291(96.29%)	1500490(2.79%)	50312801(93.5%)	3441845.0(4.89%)	1972374.0(2.8%)	58950346.0(83.7%)	1578590.0(2.24%)	4486690.0(6.37%)
M72_1	49647496(97.48%)	1146843(2.25%)	48500653(95.23%)	3599262.0(5.0%)	2005439.0(2.79%)	55679385.0(77.37%)	1401497.0(1.95%)	9277221.0(12.89%)
M72_2	53624178(97.56%)	1260129(2.29%)	52364049(95.26%)	3821517.0(4.92%)	2096476.0(2.7%)	60377269.0(77.8%)	1564452.0(2.02%)	9750551.0(12.56%)
M72_3	51171775(97.56%)	1183767(2.26%)	49988008(95.3%)	3521742.0(4.77%)	2054889.0(2.78%)	57572619.0(77.91%)	1456548.0(1.97%)	9291468.0(12.57%)
