# Supplementary material for: Methyl jasmonate elicits distinctive hydrolyzable tannin, flavonoid, and phyto-oxylipin responses in pomegranate (Punica granatum L.) leaves
Source: Planta. 2021 Sep 29;254(5):89. doi: 10.1007/s00425-021-03735-9 (PMC8481150; doi:10.1007/s00425-021-03735-9)
Supplement: Supplementary file 5 — Supplementary file5 (PDF 72 KB) [file 425_2021_3735_MOESM5_ESM.pdf]

Table S4. Length distribution of pomegranate transcripts.

Length distribution of transcripts from the 2-h, 6-h, and 24-h transcriptomes.

Length (bp)	Number of transcripts
0-1000	18,613
1001-2000	17,088
2001-3000	8,862
3001-4000	3,690
4001-5000	1,567
5001-6000	688
6001-7000	352
7001-8000	158
8001-9000	76
>9000	184
total	51,278

Length distribution of transcripts from the 72-h transcriptomes.

Length (bp)	Number of transcripts
0-1000	18,430
1001-2000	18,449
2001-3000	10,643
3001-4000	4,977
4001-5000	2,158
5001-6000	960
6001-7000	431
7001-8000	227
8001-9000	110
>9000	267
Total	56,652
